# Open government data: A systematic literature review of empirical research

**DOI:** 10.1007/s12525-022-00582-8

**Published:** 2022-09-20

**Authors:** Bernd W. Wirtz, Jan C. Weyerer, Marcel Becker, Wilhelm M. Müller

**Affiliations:** German University of Administrative Science Speyer, Chair for Information & Communication Management, Postbox 1409, 67324 Speyer, Germany

**Keywords:** Open government data, Open data, Digital economy, Digital business, Literature review, Research agenda, H1

## Abstract

Open government data (OGD) holds great potential for firms and the digital economy as a whole and has attracted increasing interest in research and practice in recent years. Governments and organizations worldwide are struggling in exploiting the full potential of OGD and require a comprehensive understanding of this phenomenon. Although scientific debates in OGD research are intense and heterogeneous, the field lacks theoretical integration of OGD topics and their systematic consideration in the context of the digital economy. In addition, OGD has been widely neglected by information systems (IS) research, which promises great potential for advancing our knowledge of the OGD concept and its role in the digital economy. To fill in this gap, this study conducts a systematic literature review of 169 empirical OGD studies. In doing so, we develop a theoretical review framework of Antecedents, Decisions, Outcomes (ADO) to unify and grasp the accumulating isolated evidence on OGD in context of the digital economy and provide a theory-informed research agenda to tap the potential of IS research for OGD. Our findings reveal six related key topic clusters of OGD research and substantial gaps, opening up prospective research avenues and particularly outlining how IS research can inform and advance OGD research.

**Supplementary information:**

The online version contains supplementary material available at 10.1007/s12525-022-00582-8.

## Introduction

In the age of the digital economy, data have become a new currency and an indispensable asset for organizations. Data constitutes the foundation of innovative technologies and applications (e.g., AI and IoT) and data-driven insights and management are vital for organizational success. The advancing digitalization in the public sector over the last decade has led to large amounts of data, making the public sector one of the main producers of data in the digital economy. A substantial part of this data pool is freely provided to the public and is commonly referred to as open government data (OGD) (Kim, 2018; Lim, 2021). As the number and worldwide development of OGD initiatives continue to advance in light of its great importance (Attard et al., 2015; Piotrowski, 2017), the widely unexplored relationship between OGD and the digital economy becomes of increasing interest.

On the one hand, the digital economy itself constitutes an important driver of OGD adoption and the successful implementation of OGD programs, as IT firms, for instance, supply public administration with mission-critical tangible (e.g., hardware and software), human (e.g., IT consultants), and intangible (e.g., IT and data know how) IT resources. On the other hand, OGD constitutes a new source of innovation and economic growth for the digital economy. OGD offers the potential to create innovation and to increase economic value sustainably - for both the public and the private business sectors. It may serve organizations as a free and meaningful complementary data source in developing new products or services, as well as in improving business intelligence, R&D, and business processes (Magalhaes & Roseira, 2020). Thus, OGD and the digital economy are characterized by a reciprocal relationship, in which both sides benefit from each other.

While the public value of OGD in terms of leveling up the transparency of governmental activities, the political participation of citizens and the collaboration between governments and external stakeholders is well-documented (Lee et al., 2019; Ruijer et al., 2017), its great opportunities and importance for the digital economy and commercial use have been widely neglected. According to the World Wide Web Foundation (2017), the impact of OGD on the economy even in the top ten countries worldwide remains rather low, averaging four out of ten on their assessment scale. A recent survey of 178 U.S. firms on the use OGD further reveals that the frequency of application varies across different forms of use, ranging from 9% (data to fact) to 44% (data to service) (Magalhaes & Roseira, 2020). These figures indicate that firms and the digital economy as a whole seem to struggle in using OGD and exploiting its full potential. This is also reflected in the current research landscape, in which the OGD concept has been predominantly examined in public administration and public management research, while receiving little attention in the field of information systems (IS) and digital business research.

Given its relevance for the digital economy and close relatedness to information systems and various associated research streams (e.g., big data analytics, AI and IoT), it is essential to frame OGD more broadly in the context of the digital economy and build a bridge to IS and digital business research. The stronger involvement of the latter promises great potential for further advancing the OGD concept and filling in the gap pertaining to its role in the digital economy and commercial use, as demanded in the literature (Magalhaes & Roseira, 2020). In order to better familiarize the IS and digital business research community with OGD and meaningfully involve it in the scholarly discussion, it is essential to first convey a broad understanding of the concept, its research landscape, and specific starting points for potential research endeavours.

As the role of the digital economy in OGD initiatives and the value potential of OGD is influenced by the antecedents of OGD programs (e.g., sophistication of governmental data infrastructures), the decisions and actions taken by the government for implementing OGD (e.g., strategic positioning and scope of governmental OGD activity), as well as the achieved outcomes and impacts (e.g., efficiency gains through and acceptance of OGD), it seems particularly promising to examine OGD and its relevance for the digital economy along these dimensions.

The research field of OGD has been on the rise over the last decade. While the number and heterogeneity of contributions are increasing, comprehensive literature reviews remain scarce in the context of open government (Tai, 2021), in particular from an IS perspective. Most importantly, OGD research lacks theoretical foundation and integration of OGD topics (Hassan & Twinomurinzi, 2018), as well as their systematic examination in the context of the digital economy. Taken together, the literature fails to provide a theoretical framework combining theoretical and empirical insights on OGD with regard to its antecedents, decisions, and outcomes, in which the concept is framed more broadly in the context of the digital economy, and which yields a research agenda that meaningfully involves the field of IS and digital business research. To fill in this gap, we conduct a systematic literature review to address the following research questions: (1) what do we know about the antecedents, decisions, and outcomes of OGD and their relation in the context of the digital economy, and (2) how can IS and digital business research inform OGD research in this connection?

To answer these research questions, the remainder of the study is structured as follows: The next section discusses definitional issues of OGD, delineating it from the closely related concepts of open government and open data. We then present an overview of prior literature reviews related to OGD and illustrate their shortcomings and implications for the study at hand. Subsequently, we describe the methodological approach and results of the systematic review of OGD literature and develop an overarching theoretical framework to integrate and synthesize thematic clusters of OGD research. Based on this, we derive a research agenda for future research on OGD providing concrete research avenues for IS and digital business research. In the final section, the findings and implications are discussed in the context of prior research and the digital economy.

### Defining open government data between the poles of open government and open data

OGD is closely related to other concepts, in particular, open government and open data. Although it may be viewed as a hybrid of both of these more general concepts (Sayogo et al., 2014), the extensive number of dedicated OGD studies in recent years indicates not only the increasing scholarly interest but also that OGD has established itself as a distinct concept and research stream separate from its superordinates, open government and open data. This also becomes apparent when looking at differentiated definitions of each concept. To begin with, open government is generally defined as “a multilateral, political, and social process, which includes in particular transparent, collaborative, and participatory action by government and administration” (Wirtz & Birkmeyer, 2015, p. 382). Although OGD can be viewed as a manifestation thereof underlying the same principles of transparency, collaboration and participation (Wirtz et al., 2019), it sets itself apart from the general concept through its data character and thus its inherently closer link to information systems.

This data characteristic is – besides the openness – the common denominator of the OGD and the open data concept and separates both from the open government concept. A widely used definition of open data refers to data that “can be freely used, modified, and shared by anyone for any purpose” (Open Knowledge Foundation, 2021). The definition of open government and its delineation from open data has been subject to many debates in the literature (Bogdanović-Dinić et al., 2014; Karkin & Yavuz, 2017; Kim, 2018; Sayogo et al., 2014). Although some earlier approaches use both terms synonymously (Janssen et al., 2012; Veljković et al., 2014), there is meanwhile consensus in the literature that OGD constitutes a subform of open data and the special distinguishing mark is that OGD is data collected by means of public funding and/or provided by public sector organizations (Borgesius et al., 2015; Kim, 2018; Lim, 2021). Accordingly, OGD is defined as “non-confidential, non-privacy-restricted data collected using public funding that is made freely available for anyone to download” (Lim, 2021, p. 1) or put more simple as “[p]ublic sector information made available to the public as open data” (Kim, 2018, p. 20). Thus, its government relatedness is the decisive element distinguishing it from open data.

For a better understanding of the scope and nature of OGD, the OECD (Ubaldi, 2013) has developed a typology of OGD, distinguishing between seven major categories: (1) business data (e.g., chamber of commerce information and official business information with regard to company or industry data), (2) registers and data pertaining to patents, trademarks, and public tenders, (3) geographic data (e.g., topographic and address data), (4) legal data (e.g., court decisions, legislation data), (5) meteorological data (e.g., weather and climate data), (6) social data (e.g., population, employment, and public health data), and (7) transport data (e.g., vehicle registrations, traffic, and public transport data). This typology underlines the particularities of OGD and indicates its various application opportunities and value for businesses.

### Prior literature reviews on OGD

The widespread scientific interest in OGD is reflected in a large number of studies, which have been the motivator and starting point for various overview studies. With a view to placing our systematic literature review in the existing field of literature reviews and determining its potential contribution to future OGD research, we first identified and analyzed the thematically relevant set of previous literature reviews. We systematically searched for literature reviews in different databases, including EBSCO (including Academic Search Premier, Business Source Premier, and EconLit with Full Text), Web of Science, ScienceDirect, ProQuest, and Google Scholar. This yielded a total of twelve dedicated literature reviews that were obtained for further analysis. To determine the scientific added value of our study, it is important to contrast the core structure and key topics of these literature reviews briefly and concisely, see Table 1.

**Table 1:** Overview of former literature reviews

Author(s) (Year)	Title / Journal	Number of Analyzed Articles	Research Focus	Contextual cluster	Core Topics
Zuiderwijk et al. (2014)	Innovation through open data: A review of the state-of-the-art and an emerging research agenda Journal: Journal of Theoretical & Applied Electronic Commerce Research	*n* = 19	Open Government Data	Cluster (3): General approaches	*General / conceptual development:* • Overview of related Institutional and organizational theories *OGD governance / regulation:* • OGD policy-making
Attard et al. (2015)	A systematic review of open government data initiatives Journal: Government Information Quarterly	*n* = 75	Open Government Data	Cluster (2): Specific OGD aspects	*General / conceptual development:* • Definition of an OGD lifecycle OGD implementation: • OGD initiatives’ components & elements
Wirtz and Birkmeyer (2015)	Open Government: Origin, Development, and Conceptual Perspectives Journal: International Journal of Public Administration	*n* = 66	Open Government	Cluster (1): OGD as side aspect	*General / conceptual development:* • OGD Definition and scope *OGD implementation / usage:* • Development of an integrative framework
Hossain et al. (2016)	State-of-the-art in open data research: Insights from existing literature and a research agenda Journal: Journal of Organizational Computing and Electronic Commerce	*n* = 96	Open Data	Cluster (1): OGD as side aspect	*General / conceptual development:* • Overview of OGD literature *OGD implementation / usage:* • OGD components & elements
Ruijer and Martinius (2017)	Researching the democratic impact of open government data: A systematic literature review Journal: Information Polity	*n* = 43	Open Government Data	Cluster (2): Specific OGD aspects	*OGD outcomes and impacts:* • Democratic impacts of OGD • Indicators to measure Democratic impacts of OGD
Safarov et al. (2017)	Utilization of open government data: A systematic literature review of types, conditions, effects and users Journal: Information Polity	*n* = 101	Open Government Data	Cluster (2): Specific OGD aspects	*OGD implementation / usage:* • Development of an OGD utilization framework *OGD outcomes and impacts:* • Impacts of OGD • Users of OGD
Criado et al. (2018)	Revisiting the Open Government Phenomenon. A Meta-Analysis of the International Literature Journal: eJournal of eDemocracy and Open Government	*n* = 189	Open Government	Cluster (1): OGD as side aspect	*General / conceptual development:* • OGD definition and scope • Demarcation of open government and open government data literature
Saxena (2018)	Summarizing the decadal literature in open government data (OGD) research: a systematic review Journal: Foresight	*n* = N/A	Open Government Data	Cluster (3): General approaches	*General / conceptual development:* • Overview of OGD literature
Haini et al. (2020)	Factors Influencing the Adoption of Open Government Data in the Public Sector: A Systematic Literature Review Journal: International Journal on Advanced Science Engineering Information Technology	*n = 25*	Open Government Data	Cluster (2): Specific OGD aspects	*OGD antecedents:* • Influence factors of OGD adoption
Purwanto et al. (2020)	Citizen Engagement with Open Government Data: A Systematic Literature Review of Drivers and Inhibitors Journal: International Journal of Electronic Government Research	*n = 52*	Open Government Data	Cluster (2): Specific OGD aspects	*OGD antecedents:* • Drivers and inhibitors of OGD citizen engagement
Francey and Mettler (2021)	The Effects of Open Government Data: Some Stylised Facts Journal: Information Polity	*n = 17*	Open Government Data	Cluster (2): Specific OGD aspects	*General / conceptual development:* • Effects of OGD
Tai (2021)	Open Government Research over a Decade: A Systematic Review Journal: Government Information Quarterly	*n = 189*	Open Government	Cluster (1): OGD as side aspect	*General / conceptual development:* • Definition and scope of OG/OGD • Overview of OG/OGD literature *OGD implementation / usage:* • Use/implementation of OG/OGD *OGD outcomes and impacts:* • Impacts/outcomes of OG/OGD

The literature reviews identified can be classified into three clusters: (1) reviews treating OGD as a side aspect, (2) reviews focusing on a specific aspect of OGD literature, and (3) reviews with a general approach towards OGD literature. The first cluster contains four out of twelve reviews identified. These reviews do not clearly distinguish between open government, open data, and OGD, and thus mix OGD studies in their analysis with those from one of the other research streams. To begin with, Hossain et al. (2016) provide a general systematization of the research field of open data, addressing OGD as one of five subareas and deriving corresponding research implications. The other three reviews in this cluster focus on OGD including OGD studies as a subset in their analyses*.* While Wirtz and Birkmeyer (2015) concentrate on the development of an integrative framework to better understand open government in general, Criado et al. (2018) attempt to explain the phenomenon of open government by means of a comprehensive analysis of existing literature and provide a comprehensive overview without deriving overly specific research implications. Likewise, Tai (2021) also provides a comprehensive review of open government research, focusing on three aspects, namely its conceptual development, its use and implementation, as well as the impacts or outcomes of open government initiatives. However, an integrated consideration as applied by the above-mentioned reviews in the first cluster confounds a clear picture of OGD research and carries the risk of arriving at undifferentiated and ultimately inaccurate conclusions. Therefore, it is essential to conduct review studies that are solely dedicated to the field of OGD, as is the case with the second and third cluster of review studies.

The second cluster is the largest and is composed of six out of twelve literature reviews identified. These reviews analyze a certain segment of OGD literature depending on a selected subtopic. The work of Attard et al. (2015) clearly focuses on the description of OGD initiatives and their respective components. They are less concerned with mapping and structuring the literature as a whole but rather with analyzing OGD initiatives and related approaches. In contrast, Ruijer and Martinius (2017) set their focus more specifically by examining literature and deriving specific research implications in relation to the democratic impact of OGD. Safarov et al. (2017) have a different emphasis by orienting their literature evaluation and systematization towards the development of an OGD utilization framework and pointing out utilization-specific research opportunities. Moreover, the literature review of Haini et al. (2020) has a special view upon studies concerning influence factors of OGD adoption in public sector organizations, identifying 16 influence factors and classifying them according to three dimensions (i.e., technological, organizational, and environmental). In contrast, Purwanto et al. (2020) focus on the citizen perspective in their review and analyze studies that deal with drivers of and barriers to citizen engagement with OGD. They identify seven groups of drivers and three categories of barriers, developing a conceptual model of citizen engagement with OGD. Finally, Francey and Mettler (2021) review case studies and examine empirical evidence on the effects of OGD, deriving nine stylized facts. While all of the studies in the second cluster provide valuable insights into the field of OGD, they only do so for the respective subtopic analyzed. None of these reviews systematizes the entire field of research and identifies the implications for further necessary research. Although Safarov et al. (2017) make a well-conceived attempt to broadly analyze and systematize based on their grouping along four key topics and the further subdivision thereof, their findings still remain specific in that they are primarily concerned with the utilization of OGD. Thus, the reviews in this cluster do not allow to make profound comparisons among subtopics within the field or to draw general conclusions in order to improve our understanding of relationships among subtopics and the state of research as a whole. This can only be achieved by reviews with a comprehensive perspective, like those in the third cluster of our literature review analysis.

This cluster is the smallest and comprises only two reviews, indicating the lack of reviews with a comprehensive focus on OGD research. These approaches are most relevant to our study because they likewise address the OGD topic as a whole. In doing so, Zuiderwijk et al. (2014) examine individual studies in relation to their topic and theoretical foundation. They offer a brief outlook on potential fields of research related to the three core topics they identified, including theory and development; policies, use, and innovation; as well as infrastructures and technology. Saxena’s (2018) systematic literature review likewise classifies OGD studies into three general clusters, i.e. theoretical and conceptual research, applied research, and user-focused research. Despite their valuable contributions both studies lack theoretical foundation and integration of the clusters. Moreover, both reviews each propose a very general taxonomy to structure research. Both taxonomies contain three clusters and appear to be little differentiated given the heterogeneity of the current research landscape. Paired with their purely descriptive nature of analysis, they only provide basic research implications that lack thematic specification and thoroughness.

The above-mentioned studies in each cluster constitute a thorough selection of OGD-related literature reviews in peer-reviewed journals. However, a literature search in the databases of AIS, IEEE, and ACM shows that several literature reviews on OGD have also been published in conference proceedings, which also should be acknowledged at this point. These contributions can also be classified according to the proposed clusters and are subject to the same shortcomings and criticism. While the broad and very early approach of Novais et al. (2013) can be assigned to the third cluster of reviews, all of the other review attempts belong to the second cluster, as they focus on specific aspects in connection with OGD, in particular, barriers or problems associated with OGD implementation and development (Bachtiar et al., 2020; Crusoe & Melin, 2018; Neto et al., 2018; Roa et al., 2019), but also challenges and opportunities associated with OGD (Hassan & Twinomurinzi, 2018), or the impact of civil servants’ behavioral factors on the opening of government data (Kleiman et al., 2020).

Overall, the analysis of literature reviews confirms the conceptual autonomy of OGD and its independent research stream (emphasized in the above-mentioned definitional considerations), since eight out of twelve reviews are specifically dedicated to OGD. Our findings further show that previous review approaches lack theoretical integration of OGD issues and do not consider them in the context of the digital economy. Accordingly, they do not provide answers to our research questions of what we know about the antecedents, decisions, and outcomes of OGD and their relation in connection with the digital economy and how IS and digital business research can inform OGD research in this respect. Given the increasing importance of OGD and the digital economy as well as their reciprocal relationship, it is essential for the further development and a better understanding of the OGD concept to systematically theorize and synthesize the respective body of knowledge. Our systematic literature review goes beyond prior literature reviews and addresses their shortcomings by developing a theoretical review framework of antecedents, decisions, and outcomes of OGD, elaborating them in relation to the digital economy and deriving a theory-informed research agenda to tap the potential of IS and digital business research for OGD.

## Methodology of the systematic literature review

### Literature selection

The literature review is based on established methodological recommendations regarding a general literature review’s overall structure and the related process of identification and selection of relevant studies (Tranfield et al., 2003; Webster & Watson, 2002). In order to comprehensively and systematically search for and select relevant studies, we followed further procedural guidelines according to the well-established PRISMA flow process adhering to its individual steps of identification, screening, eligibility, and final inclusion (Liberati et al., 2009).

To identify relevant records from established and relevant academic databases, we initially conducted a title, abstract, and subject search in different databases, including EBSCO (including Academic Search Premier, Business Source Premier, and EconLit with Full Text), Web of Science, ScienceDirect, and ProQuest*.* The search included the terms “open government data”, “data openness”, and “open data” in combination with “government” and “governance”. For the purpose of scientific rigor and quality, the search was limited to articles published in peer-reviewed academic journals in English language (Wang et al., 2019). Subsequent to the identification and elimination of duplicate records, editorial notes, and comments, the retrieved articles were first screened regarding title and abstract to determine and exclude irrelevant studies. The remaining articles were then subjected to a full-text review to exclude any studies that were not empirical and whose thematic focus was not clearly attributed to the field of OGD. This initial literature approach resulted in a total of 125 articles conforming to the selection criteria. To complement this set of literature with meaningful conference papers, we likewise searched the databases of AIS, IEEE, and ACM, yielding another 37 relevant articles. To minimize the risk of missing relevant studies, we finally screened the Google Scholar database using the same search terms with attention to the same criteria, since Google Scholar is the most comprehensive database (Gusenbauer, 2019; Martín-Martín et al., 2020) and is considered to be especially useful for identifying influential studies within specific fields of research (Martín-Martín et al., 2017; Zientek et al., 2018). In this way, seven additional eligible studies were identified and added to the selection, resulting in a final set of 169 relevant studies from the overall literature search, which represents the basis of the following preparation and analysis. Similarly to the entire selection process and assessment of eligibility, the further review, coding, and classification of the literature was performed by two reviewers. They were supported by a third reviewer who took a mediating role to assist once again in case of disagreement. The analysis of the literature consisted of two steps. The first step of our approach comprised the identification of key topic clusters in the literature by means of a bottom-up coding approach in order to determine what kind of topics are actually prevalent in the literature without constraining the result to certain areas. The second step referred to the theoretical integration of these clusters by means of a framework-based approach. In the following, we explain the methodological procedures underlying these two steps of analysis in more detail.

### Identification of key topic clusters

In this first step of the analysis, the individual studies were assigned to individual clusters according to their respective content and thematic structure. Due to the thematic complexity of OGD and the associated heterogeneity of research, as well as different foci of the individual studies, the development and final formulation of the individual key topic clusters were designed and refined through a stepwise systematic coding process. This coding process relied on the approach of Saldaña (2013) and incorporated techniques of initial coding and pattern coding. Initial coding is an open form of coding, in which qualitative information is broken down into discrete aspects. While initial coding is the first step of analysis and serves “as a starting point to provide the researcher with analytic leads for further exploration” (Saldaña, 2013, p. 101), pattern coding takes the analysis to a higher and more abstract level by refining the codes developed in the initial coding step and merging them into superordinate categories. The openness of this two-step approach already indicates that it follows an inductive procedure without a predefined coding scheme. This means that the formed concepts or categories emerge from the given data, which is characteristic for a bottom-up approach (Urquhart, 2013). Following this procedure, relevant information from the respective studies was initially coded. The resulting codes were then carefully and repeatedly examined to determine patterns in terms of similarities, correlations, and dissimilarities. The respective key topic clusters were then compared regarding their overall degree of similarity or distinction and refined, if necessary, in order to achieve optimum accuracy and consistency. This procedure yielded a final set of six key topic clusters, including (1) general/conceptual development (OGD theory), (2) drivers/barriers (OGD antecedents), (3) adoption/usage/implementation (OGD decisions), (4) success/performance/value (OGD outcomes), (5) acceptance/satisfaction/trust in government (OGD impacts), and (6) policies/regulation/law (OGD governance). The literature was then analyzed and structured according to these key topic clusters and a number of other classification criteria, including study type, method of analysis, data collection, and research perspective. The results of this step of analysis are depicted in the overview and evolution of the OGD literature.

### Theoretical integration of key topic clusters

**Fig. 1:**
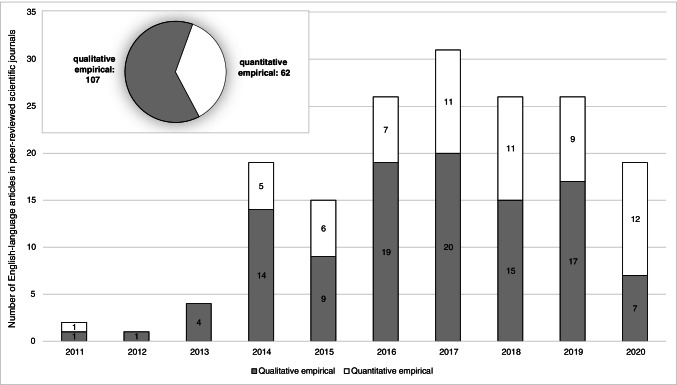
Development of open government data research

The second step referred to the theoretical integration of these clusters and thus their arrangement in a common complex of meaning. Here, we applied a framework-based approach (Paul & Criado, 2020), developing an overarching theoretical review framework that organizes the theoretical relationships among the identified thematic clusters of OGD in terms of a relationship map (Watson & Webster, 2020). This framework-based approach to literature was informed by previous literature reviews (Kessler & Chakrabarti, 1996; Lane et al., 2006; Raisch & Birkinshaw, 2008) and is particularly based on the antecedents, decisions, and outcomes (ADO) framework by Paul and Benito (2018), which is regarded as “an excellent framework to organize the findings (i.e., constructs and its ensuing relationships) of past research in a structured assembly” (Lim et al., 2021, p. 537). The ADO framework approach appeared to be particularly suitable as it provides overarching and general theoretically linked dimensions to which the specific clusters could be meaningfully assigned. Thus, the framework-based approach, i.e. the predefined dimensions of the ADO framework and their relationships given by prior literature, provides an established but at the same time only rough grid, which is specified with the core clusters identified by means of the bottom-up method in the first step of the analysis. The theoretical review framework developed in this second step of analysis and the corresponding theoretical integration of the core clusters in the context of the digital economy are presented in the synthesis of OGD literature. The framework finally also serves as a point of reference for deriving the theory-informed research agenda for IS and digital business research (Fig. 1).

## Overview and evolution of the OGD literature

To provide a better understanding of the extent and evolution of the empirical OGD literature, this section gives a brief overview of its general development and current state. To begin with, Fig. 1 illustrates the distribution of qualitative and quantitative empirical OGD studies over the last 10 years.

Considering that OGD has evolved as an independent research stream out of general open government and open data research, it is not surprising that empirical research on OGD developed with a certain time lag in comparison to both of these more general research streams. Although OGD-related research was initially, in particular, an integral part of open government research, the first empirical and dedicated OGD studies appeared in 2011. Academic interest has increased significantly since 2014 and, measured by the number of empirical studies, of which a total of 107 (about 63%) studies are of a qualitative and 62 (about 37%) are of a quantitative design, remains high. The peak in 2016 and 2017 is due to a comparatively greater number of pertinent conferences and respective publications in these years. The decline in 2020 may be a result of the coronavirus pandemic, which has disrupted and delayed research projects and funding in general (Callaway et al., 2020).

**Fig. 2:**
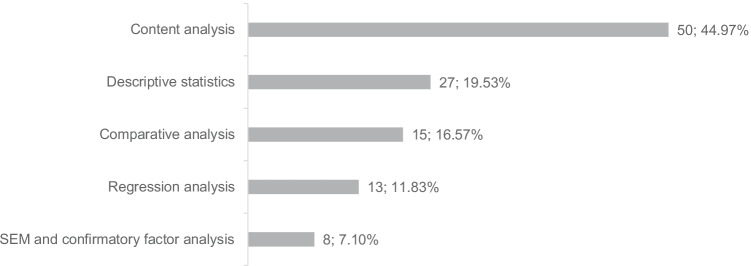
Number of studies according to applied method of analysis

Corresponding to the allocation of qualitative and quantitative empirical studies, the majority of the studies apply qualitative content analyses based on either an individual or comparative approach (61.54%). The application of quantitative methods is consequently lower in total, whereby publications using methods of complex empirical research, such as regression analysis and structural equation modeling, with a combined share of 18.93%, number even fewer, as opposed to publications based on descriptive statistics (19.53%). Figure 2 depicts the distribution of the applied methods of analysis.

Table 2 presents the identified key topic clusters and provides selected descriptive statistics how these key topics have been approached in terms of study type, data collection, and research perspective.

**Table 2:** Overview of classification criteria and descriptive statistics of the literature review

(A) Classification criteria:				
Key topic	Study type	Method of analysis	Data collection	Perspective
* OGD theory:* general/conceptual development	Qualitative	Content analysis	Interview	Provider
* OGD antecedents:* drivers/barriers	Quantitative	Comparative analysis	Questionnaire	User
* OGD decisions:* adoption/usage/implementation		Descriptive statistics	Secondary data	Mixed
* OGD outcomes:* success/performance/value		Regression analysis	Mixed methods	
* OGD impacts:* acceptance/satisfaction/trust in government		SEM / confirmatory factor analysis		
* OGD governance:* policies/regulation/law				
(B) Study type of reviewed studies according to key topic:				
Key topic	Qualitative	Quantitative	Total	Share
* OGD theory:* general/conceptual development	12	7	19	11,24%
* OGD antecedents:* drivers/barriers	17	10	27	15,98%
* OGD decisions:* adoption/usage/implementation	30	18	48	28,40%
* OGD outcomes:* success/performance/value	31	18	49	28,99%
* OGD impacts:* acceptance/satisfaction/trust in government	1	7	8	4,73%
* OGD governance:* policies/regulation/law	14	4	18	10,65%
Total	105 (62.13%)	64 (37.87%)	169 (100%)	100%
(C) Method of data collection according to key topic				
Key topic	Interview	Questionnaire	Secondary data	Mixed methods
* OGD theory:* general/conceptual development	2	1	7	9
* OGD antecedents:* drivers/barriers	3	4	12	8
* OGD decisions:* adoption/usage/implementation	3	12	24	9
* OGD outcomes:* success/performance/value	1	4	33	11
* OGD impacts:* acceptance/satisfaction/trust in government	0	3	2	3
* OGD governance:* policies/regulation/law	2	0	12	4
Total	11 (6.51%)	24 (14.20%)	90 (53.25%)	44 (26.04%)
(D) Number of studies according to key topic and research perspective				
Key topic	Provider	User	Mixed	Total
* OGD theory:* general/conceptual development	8	2	9	19
* OGD antecedents:* drivers/barriers	17	2	8	27
* OGD decisions:* adoption/usage/implementation	22	12	14	48
* OGD outcomes:* success/performance/value	32	6	11	49
* OGD impacts:* acceptance/satisfaction/trust in government	0	8	0	8
* OGD governance:* policies/regulation/law	18	0	0	18
Total	97 (57.40%)	30 (17.75%)	42 (24.85%)	169 (100%)

Table 2 shows that the largest share of the research focuses on the key topic (4) OGD outcomes and accounts for 28.99% of the literature reviewed, which is not surprising given the extensive impact of OGD on different performance and success levels. The key topic, with an almost equal number of assigned studies, is the group (3) OGD decisions with 28.40%, followed by the key topics (2) OGD antecedents with 15.98%, and (1) OGD theory with 11.24%. While the share of studies in key topic (6) OGD governance remains in the double-digit percentage range (10.65%), the level of scientific interest measured by the number of publications within key topic (5) OGD impacts is significantly lower (4.73%). Furthermore, like the overall distribution of qualitative and quantitative empirical research occurs the composition with regard to the individual key topic clusters, so that the number of qualitative studies clearly predominates in each key topic. Notably, key topic (5) OGD impacts constitutes an exception, where the exact opposite is the case. This pattern can be explained by the fact that research on OGD is still at a relatively early stage.

In summary, the analysis reveals the great scope and heterogeneity of the research landscape of OGD in terms of research focus and methodology. The pronounced imbalance between qualitative and quantitative studies in favor of the former indicates that OGD is still an emerging field of research. Given this emergent state of research, quantitative empirical studies are essential to confirm causality of theoretical relationships and effects of evolving issues proposed by conceptual or qualitative research, and to address associated concerns of validity. In particular, little empirical robust knowledge is available in the areas of acceptance/satisfaction/trust in government, policies/regulation/law general/conceptual development, and drivers/barriers. This also holds when it comes to understanding the user perspective in the context of OGD, which is generally neglected in the field, but in particular in these areas. A remarkable exception to this pattern is the area of acceptance/satisfaction/trust in government, which has so far only focused on the user perspective, while disregarding the provider perspective. However, this would be especially important in view of the struggling implementation and diffusion of OGD in several public organizations. The user perspective so far has also strongly emphasized the individual level (e.g., citizens) and should increasingly consider the organizational level (e.g., firms) for a better understanding of the role of OGD in the digital economy.

## Synthesis of the OGD literature

**Fig. 3:**
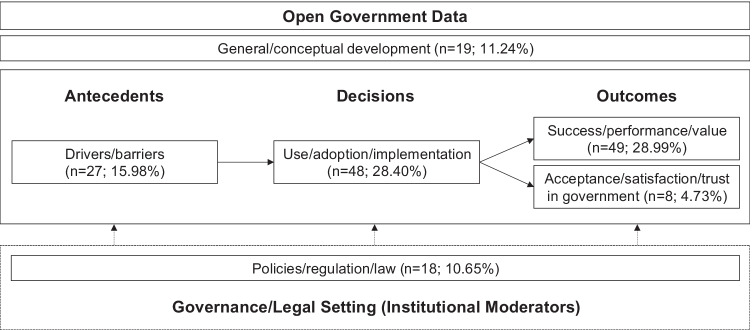
Overarching theoretical review framework

The synthesis of the OGD literature is based on the theoretical review framework and theoretically integrates the previously identified key topic clusters with reference to the digital economy. Figure 3 depicts the review framework and the theoretical relationships among the identified key topic clusters.

The framework may serve as a thematic relationship map of empirical OGD research, particularly illustrating the associations among antecedents, decisions, and outcomes of OGD, as well respective focus areas of research and neglected topics. The antecedents in terms of the drivers and barriers explain the reasons for a certain behavior, while decisions determine the forms of behavior (i.e. adoption, usage, or implementation of OGD), and outcomes comprise the assessments that result from decisions and the associated behavior (i.e., success, performance, and value or acceptance, satisfaction, and trust in government) (Lim et al., 2021). All these processes take place in a governance and regulatory setting, in which policies, regulation, and law may affect this process in terms of institutional moderators. These layers underlie the general and conceptual development of OGD, which is the overarching object of action and knowledge, and thus constitutes the point of reference for all other elements in the framework. The synthesis of OGD literature is conducted along these dimensions in the following.

## General conceptual development of OGD

### Perspectives on OGD

Regarding the general conceptual development of ‘Open Government Data’, various studies contrast four ways of perceiving the term in recent years (Alexopoulos et al., 2018; Gonzalez-Zapata & Heeks, 2015; Jetzek et al., 2013): (1) the bureaucratic perspective conceiving OGD as a bureaucratic mechanism to enhance information quality, effectiveness and efficiency of government policy making, and legitimacy of polices (cf. Alexopoulos et al., 2018; Gonzalez-Zapata & Heeks 2015), (2) the technological perspective conceiving OGD as a technological innovation of public administration building up a data infrastructure to host a freely available public database of accurate, complete, and timely public sector data (cf. McNutt et al., 2016; Meijer, 2015), (3) the political perspective conceiving OGD as a part of government accountability to the citizens, thus providing insights into government affairs, transparency of governmental action, and the option for civic participation in policymaking (cf. Zhao & Fan, 2018; Meijer, 2015), and (4) the economic perspective conceiving OGD as source of economic value creation, providing several opportunities for the commercialization of these data in new goods and services (cf. McBride et al., 2019; Zhao & Fan, 2018; Berrone et al., 2017).

### The digital economy’s role in the OGD ecosystem

**Fig. 4:**
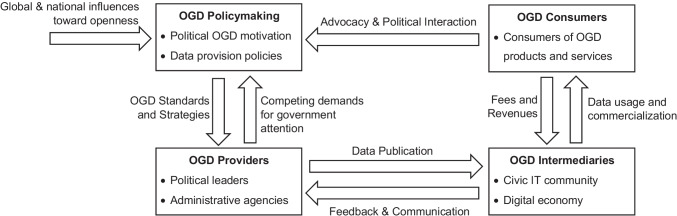
Open government data ecosystem (based on Dawes et al., 2016 and Kassen, 2013)

Against the background of the OGD ecosystem model presented by Dawes et al. (2016), these perspectives of the literature can be interpreted as to portray four fields of stakeholder interactions in OGD settings. In this context, the bureaucratic perspective focuses on the interaction between the policymakers and the implementing authorities by surveilling the effects (increase in the quality of information, the effectiveness of administrative action, the legitimacy of public policy) (cf. Alexopoulos et al., 2018), while the political perspective regards OGD as a means for democratic processes and decision-making, as it investigates the role of OGD in government accountability, transparency, and citizen participation. Correspondingly, the technological perspective portrays the interaction between OGD providers (public authorities) and OGD intermediaries (i.e., the digital economy) by stating the technical characteristics of the data infrastructure. The economic perspective, however, lays its focus upon the creation of value for the OGD customers, i.e., the citizens, by investigating how OGD yields public value to them. Against this background, firms of the digital economy assume an intermediary function matching technical data supply from the government with information demand of the OGD customers. In a nutshell, the task of digital firms in the OGD ecosystem is to access the data supplied by the government, to gather the information contained in OGD by electronic data processing and analytics software, and to commercialize this information in their products and services. Figure 4 outlines the OGD ecosystem and sketches the role of the digital economy as a data intermediary facilitating the interaction between the executive government authorities and the citizens.

### Scope of government activity and the digital economy

Besides the general role of the digital economy, both the scope of digital business opportunities and the business approach are crucial to the digital economy. In this context, the literature raises interesting points regarding the scope of government activity in data-based service provision. Some studies find evidence for governments simply providing public data and setting the legal and technical framework by data formats and access rights, while leaving further processing and marketizing of these data completely to interested stakeholders, like NGOs, companies, or private citizens (cf. Alexopoulos et al., 2018; Berrone et al., 2017; Dawes et al., 2016; McNutt et al., 2016). However, another strand of OGD literature finds more complex forms of governmental open data platforms, providing data via APIs and data-based apps that enable the user to filter and manipulate the chosen data set and to embed the data in other data processing programs (cf. McBride et al., 2019; Zhao & Fan, 2018; Berrone et al., 2017). In this case, government provides OGD products and services on its own in competition to possible private sector offerings. In this context, contemporary OGD research presents a spectrum of government involvement in the presentation and processing of publicly accessible data by presenting diverging roles of government in OGD programs, i.e. data provision and standard-setting versus data service platform hosting. Consequently, the scope of government activity and the sophistication of governmental data infrastructures for the compilation, analysis, and provision of public sector data significantly influences the economic margin and targets of digital private business with OGD. Besides the theoretical setting of the digital economy’s role in the OGD ecosystem, the antecedents of OGD programs, the decisions and actions taken by the government for OGD implementation, as well as the achieved outcomes and impacts also determine the position of the digital economy in OGD programs and how to create value from public sector data.

The following subsection provides a synthesis of the findings of previous research on the antecedents, decisions, and outcomes of OGD with special reference to the digital economy, elaborating their significance for IS and digital business research (Table 1 in the online Appendix summarizes these findings). The representative studies presented in the following subsection (and in Table 1 in the online Appendix) were selected due to their high resonance in scientific research (high Google Scholar citation score) and their publication in particularly influential scientific, peer-reviewed journals (high journal impact score).

## Antecedents, decisions, and outcomes of OGD and the digital economy

### OGD antecedents: Drivers and barriers

When considering the antecedents and determinants of OGD programs, previous studies more often refer to barriers emerging from the OGD ecosystem (cf. Barry & Bannister, 2014; Janssen et al., 2012; Ruijer et al., 2017), rather than the drivers and enablers (cf. Young, 2020; Zhenbin et al., 2020; Susha et al., 2015). For the factors triggering or fostering OGD policies, the findings of previous studies distinguish among political and social factors, operational and technical properties of agency equipment, or economic opportunities for OGD usage. In case of political and social OGD determinants, political and social demand for transparency and accountability (Barry & Bannister, 2014; Janssen et al., 2012; Zhenbin et al., 2020) is perceived as a major trigger for OGD programs alongside with increasing citizen engagement and participation in government affairs (Young, 2020; Welch et al., 2016). Regarding the operational and technical drivers, previous studies highlight the importance of a cultural anchorage of electronic data processing and sharing in public administration (Zhenbin et al., 2020, Yang et al., 2015) in combination with a well-developed data infrastructure within the agency operated by qualified specialists (Young, 2020; Welch et al. 2016). In this context, economic pressure arises from a large share of private companies providing public services to the citizens for profit. Studies such as Young (2020) find that the opportunity to augment extant or create new public services by using public sector data bears opportunities to create new sources for economic growth (cf. Young, 2020; Zhenbin et al., 2020; Susha et al., 2015). This is even more the case if the national economy possesses the resources for exploiting the information contained in public sector data (high GDP) and exhibits a large productivity in providing ICT services (high share of the IT industry) (cf. Young, 2020; Susha et al., 2015). In this context, the state of the digital economy as well as the maturity of governmental data infrastructures appear as drivers for both the successful implementation of OGD programs and the successful exploitation of these data in public services. Consequently, IT firms thus function as software and hardware suppliers to public administration in digitally underdeveloped economies, while they assume the role of a private sector competitor in the delivery of public services in digitally advanced countries.

Barriers to implementing an OGD policy emerge from problems with (1) data compilation on the part of the government or the executive agencies (institutional constraints), with (2) data access caused by technical failures or dysfunctional data portals (technical constraints), or with (3) data application on the part of the citizens (societal barriers). Accordingly, data compilation barriers refer to factors that hinder the respective agencies to collect, compile, or transfer suitable data due to legal constraints (Yang et al., 2015; Barry & Bannister, 2014), due to the complexity of the organizational structures of government agencies (Ruijer et al., 2017; Welch et al., 2016; Yang et al., 2015), and/or due to the lack of their data management capacities and capabilities (Ruijer et al., 2017; Young, 2020). In contrast, data access barriers emerge from the properties of the data infrastructure. Major impediments in data access arise from a lack in system interoperability if governmental software and data formats are not compatible with its civic counterparts (Smith & Sandberg, 2018; Barry & Bannister, 2014) or from a lack in technical support and constant updating of data platforms due to staff shortages (Janssen et al., 2012). Furthermore, the literature also finds that the introduction of registered access to public data creates another great obstacle for OGD as most people are unwilling to register officially on public data platforms for occasional data access (cf.Barry & Bannister, 2014 ; Ruijer et al., 2017). Regarding the obstacles emerging from the properties of the user, i.e. the citizens, previous research argues that the success of OGD programs is to be attached to the ability of society to make use of the published data. Obstacles emerge from the inability of the users to achieve a practical use of these data; this might either be due to the societal inability of information processing (e.g., low ICT equipment, low levels of education, low income, etc.) (Barry & Bannister, 2014; Ruijer et al., 2017), or due to the uselessness of the provided data such that the citizens cannot apply the information to achieve any value (Smith & Sandberg, 2018; Janssen et al., 2012). Considering these findings, all barriers provide starting points for digital business to step in and solve the issue. In case of data compilation constraints, IT firms adapt solutions from private sector products and services to provide a customized data infrastructure to public authorities aiming to publish their data. To overcome data access barriers, private IT firms host government data for public retrieval as business partners of public authorities and provide the information via their own data services and applications. Finally, to solve data application barriers, the digital economy provides IT specialists and data analysts processing government data and create a useful summary and analysis of OGD for the citizens.

### OGD decisions: Adoption, usage, and implementation

Although the relevant drivers and obstacles open corresponding business opportunities for the digital economy, actual policy decisions regarding the adoption of OGD measures, as well as their implementation and subsequent use, are of crucial importance for business practice. As stated before, government activity in providing data-based applications to its citizens is of major importance for the type of digital business. Accordingly, previous research analyzed the decisions regarding OGD policy and strategy as well as the intensity of governmental OGD activities (Gascó-Hernandez et al., 2018; Dawes et al., 2016). Depending on the scope of governmental data processing and data-based service provision, Dawes et al. (2016) propose a spectrum of OGD policies presenting three archetypes of OGD strategy, starting with (1) the data-oriented OGD policy aiming at the provision of accurate, unbiased datasets from public sector entities without any further service features (cf. Wang & Lo, 2016; Yang & Wu, 2016), followed by (2) the intermediate program-oriented OGD policy providing public data via an OGD platform displaying basic data analysis features and APIs (cf. Chatfield & Reddick, 2017; Parycek et al., 2014), ending up with (3) the use- and user-oriented OGD policy focusing on the creation of public value by embedding public sector data within data-based public services (Gascó-Hernandez et al., 2018).

Despite these strategic considerations, governmental adoption decisions also have a major impact upon the organizational and technical preparations to get public administration ready for OGD (Chatfield & Reddick, 2017; Yang & Wu, 2016; Parycek et al., 2014). Closely connected to the strategic setting is the scope of publication permissions from high-level authorities ranging from data publication restrictions to the support of interactive data services. Furthermore, the government’s adoption decisions also shape the maturity of the authorities’ data infrastructure by defining the technical capacity as well as the interoperability and connectivity to citizen devices (Bonina & Eaton, 2020; Wang & Lo, 2016). Consequently, the ex-ante decisions regarding the adoption of OGD measures also define the way of doing business with OGD. In this regard, the strategic positioning of governmental OGD activities directly determines the scope of the intermediary role of the digital economy. In case of a data-oriented OGD program relying upon a mediocre public data infrastructure, the intermediary role of the digital economy achieves its climax as the government acts as a proper data provider, leaving data analysis, application, and embedment in public services completely to digital firms. However, privatization of data-based public services diminishes if the OGD program place special emphasis upon the user. For a user-oriented OGD program equipped with a well-developed public data infrastructure, utilizing OGD for providing data-based public services is completely in the hands of the government, whereas IT firms provide IT expertise and software solutions to the authorities.

Besides the determining character of ex-ante decisions for digital business with OGD, the ex-post decisions of the government flanking the OGD program also provide opportunities for the digital economy. Linked to the strategic setting of the OGD program is the decision for the target group and user profile of the program (Smith & Sandberg, 2018; Parycek et al., 2014). Depending on the respective policy intensity, government must decide whether (1) to grant general access for the average citizen in case of a user-oriented approach, or (2) to grant licensed commercial access enabling the embedment of OGD in the products and services offered by private IT firms in case of a program-oriented OGD approach, or (3) to grant access only to IT specialists for retrieving information via data analytics in case of a data-oriented OGD approach.

Furthermore, previous research also investigated the ensuing decisions concerning the interface design and the related features of OGD portals (Wirtz et al., 2019; Chatfield & Reddick, 2017). Accordingly, OGD portals diverge in the scope of the provided datasets, in the scope of the OGD interface as well as the scope of data service functions, ranging from mere data downloads from government websites to data service hubs created by OGD platforms. As a result, the user profile targeted by the OGD program as well as the design and features of the OGD interface shape business approaches for OGD. Accordingly, IT firms seek to gather, process, and capture value by commercializing OGD in products and services for the citizens in case of a licensed access and a low scope of OGD data service features, responding to the demand of proper data processing on the demand side of the OGD ecosystem. In case of limited specialist access and a high scope of data service functions, IT firms switch towards offering data analytics services to the authorities involved, equivalently responding to the demand of supply-sided data processing and analytics (cf. Bonina & Eaton, 2020).

Another relevant field for government decisions flanking the implementation of OGD programs refers to the creation of IT skills and technical expertise required for data management by public authorities (Gascó-Hernandez et al., 2018; Wirtz et al., 2019; Yang & Wu, 2016). Regarding the timescale and the addressees of these measures, current research distinguishes between short- to mid-term educational measures for public employees developing OGD skills and capabilities (cf. Safarov, 2019; Yang & Wu 2016) and long-term educational measures, increasing common IT knowledge among the population (cf. Gascó-Hernandez et al., 2018; Wirtz et al., 2018). Short- to mid-term OGD skill development is associated with a variety of options, ranging from internal IT trainings with the respective authorities (Yang & Wu, 2016) to joint ventures with the digital economy (Safarov, 2019). This decision area thus offers several linkages to digital business, spanning from the provision of training programs for public administration to learning-on-the-job in collaborative partnerships for OGD processing and evaluation. Regarding long-term public IT schooling, the government aims at building up IT skills and capabilities among the population in order to gain skilled employees for public administration (cf. Gascó-Hernandez et al., 2018). As a result, private-sector IT companies sell their know-how and IT expertise to educational institutions as mentoring partners for IT practice. All in all, the digital economy assumes the role of a catalyst in the field of digital education and training of the people - as trainers and administrative partners in the short term and as mentors in the long run.

### OGD outcomes: Success, performance, and value

Finally, it is of crucial importance not only to the government and public administration whether an OGD program pays off in terms of efficiency, citizen satisfaction, and trust in government. For the digital economy, the question is whether accessing and utilizing OGD provides access to new products and services as well as whether OGD can create new markets for data-based public services. Regarding the outcomes achieved by OGD implementation, most studies refer to the internal effects upon the performance of public administration, such as efficiency gains in administrative procedures and public service provision (Mergel et al., 2018; Worthy, 2015), transparency of political decisions and policy-making (Wang & Shepherd, 2020; Marjanovic & Cecez-Kecmanovic, 2017; Jetzek et al., 2014), or behavioral effects upon public employees (Marjanovic & Cecez-Kecmanovic, 2017; Worthy, 2015).

In contrast to these specific administrative and political issues, some studies also refer to spill-over effects upon the interaction of citizens with public authorities (interaction effects), the distribution of information among the population (information effects), as well as the innovation of public services by utilizing OGD (commercialization/innovation effects). Considering interaction effects upon the participation and involvement of citizens into public affairs, previous studies observe a positive effect in citizen engagement in case of OGD programs. Although there is evidence of negative OGD effects upon the polarization in political debates due to different interpretations of government data (cf. Worthy, 2015), most studies report positive effects, such as public service innovation through co-creation with citizens and IT firms or synergy effects due to simplified data sharing in collaborations between government agencies and external service providers (Ruijer & Meijer, 2020; Máchová & Lněnička, 2017; Jetzek et al., 2014). Having this mind, interaction effects of OGD programs enable the digital economy to serve as a moderator, facilitating the interaction between government and citizens by easing information processing on the part of the citizens and communication to the citizens on the part of public administration. Furthermore, IT firms relying upon big data analytics might experience competitive advantages in comparison to their international competitors as the cost for gathering public sector data decreases significantly. Consequently, citizen engagement and data sharing provide economic growth potentials to the digital economy. This is also in line with the commercialization and innovation effects observed by several studies (Jetzek et al., 2019; Mergel et al., 2018; Jetzek et al., 2014). Accordingly, previous research finds evidence for OGD spillover effects to the private sector, as implementing OGD enables digital firms to access new information at lower cost, and to generate a footage in the public sector by developing new markets for data-based products and public services.

### Acceptance, satisfaction, and trust in government

Considering the consequences on technology acceptance and citizen satisfaction triggered by OGD, previous research observes a positive impact fostered by several preconditions. In case of technology acceptance, studies find that a positive impact relies upon (1) sufficiently intense Internet usage among the population (Gonzálvez-Gallego et al., 2020; Afful-Dadzie & Afful-Dadzie, 2017), (2) the awareness of individual benefits that emerge when using and applying OGD (Zuiderwijk et al., 2015; De Kool & Bekkers, 2014), and (3) the degree of OGD usage obligation in G2C interactions (Gonzálvez-Gallego et al., 2020; Zuiderwijk et al., 2015). Considering citizen satisfaction, broad acceptance and public support of OGD and its application appear as necessary conditions alongside with a sufficiently high information quality, system quality, and service quality (cf. Gonzálvez-Gallego et al., 2020). Hence, the maturity of a country’s digital economy directly moderates the impact of OGD on technology acceptance and citizen satisfaction. This is due to developed digital economies displaying both a widespread use of ICT devices and their intensive usage, as well as common IT knowledge among the people. In addition, resident digital firms are in a much better position to support a well-functioning public data infrastructure in the case of an advanced IT industry.

In summary, it can be stated that from the perspective of public administration, the digital economy constitutes both a driver of OGD adoption and a warrant for successfully implementing an OGD program. From the perspective of the digital economy, however, OGD represents a new source of economic growth and business model innovation based upon the development of new resources, i.e., public sector data, and new business opportunities emerging during OGD adoption and implementation.

## Research agenda

The preceding identification of OGD key topic clusters and their synthesis into a theoretical framework with special reference to the digital economy has revealed significant points of connection to IS and digital business research and enables us to develop a theory-informed research agenda for the latter. Although the prior literature review emphasized particularly the core dimensions of the ADO framework, the findings also yield implications for the key topics (1) OGD theory and (6) OGD governance.

### (1) OGD theory: General/conceptual development

As the OGD ecosystem theorizes that firms of the digital economy assume an intermediary function matching technical data supply from public authorities with the demand for information on the part of the citizens, empirical research needs to verify how this assumption holds true in practice. Furthermore, future research needs to clarify the impact of government activity and OGD infrastructure maturity upon the business models of related IT firms. Consequently, McBride et al. (2019) postulate the need for further empirical research, which would enable comparison and differentiation of individual OGD services in their emergence, orientation, and goals. McBride et al. (2019) consider this especially with regard to data platforms and OGD services, which increasingly evolve from different sources. This corresponds with the implications pointed out by other researchers who identify further needs for empirical research on the characteristics of OGD sources in connection with different national contexts (Alexopoulos et al., 2018), data platforms collaboratively developed in joint ventures with IT firms (Meijer & Potjer, 2018), and the changes in the OGD portals’ datasets over time (Di Wang et al., 2018). Hence, more empirical research is needed, especially case studies regarding the economic OGD perspective, to determine the scope of involvement of private IT firms in OGD programs in general as well as their function within the whole OGD ecosystem in practice. Despite that, the scope of government activity in data-based service provision needs further investigation regarding its impact on the business approach of the digital economy. Consequently, the following questions may guide further research in this direction: How are firms of the digital economy involved in contemporary OGD programs? What is the function/business of digital IT firms in respective OGD programs? How does the scope of governmental OGD activity alter the business model of digital firms?

### (2) OGD antecedents: Drivers/barriers

Considering external OGD drivers and barriers, the preceding analysis of OGD research revealed the productivity of the IT industry, as well as the GDP share of the digital economy as key drivers of successful OGD programs. Thus, establishing a causal linkage between the size of the IT industry, the share of the digital economy, and the maturity of OGD programs appears as a suitable goal for further empirical research. Linked to this idea is also the idea of Shao and Saxena (2019) raising the question of how a society’s cultural characteristics and traditional values act as drivers and/or barriers to the intentions of administrative implementation and the participation of external actors within OGD initiatives. Consequently, the following research questions appear as a good starting point for analyzing OGD drivers and barriers emerging from the digital environment: Does a high productivity of IT firms and large share of the digital economy increase the success of OGD initiatives? Which socioeconomic, demographic, and cultural characteristics of the economy drive or impede OGD implementation?

Turning towards drivers and barriers from inside public authorities, Zhenbin et al. (2020), for instance, name the need to further investigate which specific drivers influence the motivation of government agencies to engage in OGD development and public service innovation. This has been similarly formulated by Fan and Zhao (2017), who, in addition to examining the question of which influences generally exert pressure on the internal, organizational orientation in relation to OGD activities, also emphasize the need for further research on the extensive influence of the media. With regard to policy constraints, Young (2020) identifies the risk within public institutions of intentionally withholding data/information that could be detrimental to the publisher and postulates the need to investigate more closely the existence of these barriers and their potentially negative consequences in the future. Considering the findings from the qualitative literature synthesis, the question arises as to whether collaboration with private IT companies results in a reduction of barriers or an activation of drivers within the agency. This could be empirically determined and investigated in particular by means of interviews and questionnaires. Possible research questions in this direction would be: To what extent do data access, data processing, and data application in public services improve due to collaboration with private IT companies? To what extent do intensive G2B interactions regarding OGD contribute to its successful implementation?

### (3) OGD decisions: Adoption/usage/implementation

While synthesizing the findings of previous studies, it became clear that the strategic positioning in OGD adoption, the target groups for OGD usage, as well as the organizational OGD readiness for OGD implementation have a significant impact on the orientation of the corresponding OGD business models. In light of these findings, two promising directions of research emerge for the IS research community investigating OGD in the context of the digital economy: (1) the empirical verification of the assumed correlation between the user-orientation of governmental OGD initiatives and the predominant customer alignment of IT firms’ OGD business models, and (2) the case-study-based investigation of the causal relationship between OGD access barriers and the share of the digital economy in providing data-based public services. Overall, the need for further, user-focused research is obvious and acknowledged. For example, there is a need to identify the types of datasets users of OGD require in order to enable even more active participation and usage (Chorley, 2017) and to understand how external users can be motivated to become permanent participants in OGD, while respecting their job situation and other cultural influences (Hermanto et al., 2018). Smith and Sandberg (2018) also point out that instead of the usual data-centric research, more user-centric OGD research is needed in future. In this context, the established theories of IS and digital business research such as the Technology Acceptance Model (TAM), the Unified Theory of Acceptance and Use of Technology (UTAUT), and the DeLone-McLean IS Success Model become particularly important for the further development of this field of research. Accordingly, the following research questions may guide scholars in conducting further research concerning the OGD adoption and usage approaches: How can IS theories and explanatory models, in particular, the TAM, UTAUT, and IS Success Model be applied in the context of OGD research and theory development to explain acceptance, adoption and usage behavior? How does governmental customization of OGD alter the value proposition and customer composition of OGD business models? What is the impact of OGD access restrictions on the business practices of the IT firms involved?

Another interesting avenue for further research connecting OGD to IS and digital business studies is the topic of building up relevant OGD skills and educational support. The findings from the literature synthesis suggest the digital economy to serve as a catalyst in digital education providing skills and knowledge in the short run, and innovative spirit and educational support in the long run. In this respect, Safarov (2019) points out that it might be useful to examine in more detail the design and impact of various OGD activities, such as open data awards or specific training programs. Several other researchers also discuss the necessity and value of findings based on integrative methods and trainings regarding the implementation and usage of OGD. In this way, among other things, experimental studies can be performed to determine which training methods can be used most successfully in relation to specific content and data sets in order to ensure a lasting curiosity and interest in OGD (Gascó-Hernández et al., 2018).

Further long-term studies will also show how government institutions’ perceptions and usage behavior change over time as the methods are compared (Altayar, 2018; Wang & Lo, 2016). Wirtz et al. (2018) postulate the need for further research to examine the degree to which the usage behavior of citizens changes over time and which situational and socio-cultural aspects play a role in this process. In this respect, in addition to the use of longitudinal studies, comparative cross-cultural or cross-country studies can also be used to identify relevant differences and investigate their consequences for user behavior (Saxena, 2018). Considering these demands for further research, the following research questions may inspire research regarding the role of the digital economy in creating digital OGD literacy: Do G2B partnerships in OGD increase the digital literacy of public employees? Do OGD training programs and educational measures have a greater effect on the trainees if education involves cooperation with IT firms?

### (4) OGD outcomes: Success/performance/value

Since current research on OGD outcomes is concerned with the question of how OGD offers socioeconomic added value to society, there are also potential spin-offs for the digital economy in this context. In the preceding literature synthesis, it became clear that the establishment of OGD programs could generate spillover effects on the competitiveness and innovative strength of the digital economy. Accordingly, the empirical investigation of these effects by means of case studies and time series analyses appears to be a promising goal for further research. Specifically, the following research questions suggest themselves in this context: How does the successful implementation of OGD initiatives affect the competitiveness of IT firms? Is there evidence for a causal relationship between the implementation of OGD programs and economic growth in the digital economy?

However, answering these specific research questions depends largely on the ability to record and evaluate the performance and resultant success of OGD activities. Since the success of OGD activities to be determined or measured extends to many areas among public institutions and external stakeholders, it is generally difficult to comprehensively classify and evaluate success and failure. In response to the challenges posed by the above-mentioned reasons, Marmier and Mettler (2020) postulate the need for additional research on the level of dedicated quality measurement and evaluation of OGD and its measurement instruments. Similarly, Jetzek et al. (2019) argue that the answer to the question of how data constructs and their quality are to be measured at the societal level poses another future research need.

Another relevant issue involves the potential value contribution of OGD and describes the need for further research to identify the potential contribution of OGD activities in terms of overall value creation in terms of social, economic, and public value. The origin of this value creation lies in the fact that data from public institutions are first made available in an appropriate quality, wherefrom Luna-Reyes et al. (2019) derive the need for further research to identify suitable governance and leadership approaches and to examine their influence on the quality of the data to be emitted. Mergel et al. (2018) further emphasize the large amount of valuable innovations that can be triggered by OGD and point out the need for further research in this regard to broaden and strengthen existing knowledge. Magalhaes and Roseira (2020) present similar points and show that, albeit the increasing recognition of the potential value for the private business sector, the reasons for or against integrating OGD into business processes, and thus also the potential economic value that can be achieved, still often remain unexploited or even unclear. They emphasize the need for further in-depth analysis at the firm level in order to move from a general top view to explicit insights into the behavior of and consequences for firms in their interactions with OGD. A research question of central importance might consequently be: How can dedicated products and processes be explored and exploited in order to generate sustainable economic and public value in different OGD contexts?

### (5) OGD impacts: Acceptance/satisfaction/trust in government

The synthesis of the existing literature on the topic of the consequences and impacts of OGD programs on the general acceptance of OGD, the satisfaction of citizens with its use, as well as the resulting trust in government policy suggests that these impacts are all the stronger in case of a well-developed digital economy. As argued above, this is due to (1) widespread usage of ICT devices among the population, (2) IT-related customer preferences and usage perceptions, and (3) technical support from private IT firms. Taking this implication as a starting point for further research raises the following questions: Does the maturity of the digital infrastructure moderate OGD acceptance and user satisfaction? Do joint ventures of government and private IT firms providing OGD services to the public increase trust in open government?

Against this background, the investigation of external stakeholders’ perceptions and preferences is of central importance and determines the need for further research to explore and scrutinize the differing perceptions and preferences of various stakeholders in terms of OGD activities and outcomes by international comparison. Further research efforts should therefore be undertaken to examine and compare preferences and perceived satisfaction at both the citizen (Saxena & Janssen, 2017) and corporate levels (Afful-Dadzie & Afful-Dadzie, 2017). Due to the small number of studies dedicated to OGD impacts, it is of interest to broaden the focus from the external stakeholders to an in-depth investigation of the acceptance and satisfaction of governmental agencies’ internal forces, as these act as a starting point or barrier to subsequent external perception and satisfaction (Barry & Bannister, 2014). Consequently, scientific progress within the field of OGD antecedents might also spark research efforts in OGD impacts.

### (6) OGD governance: Policies/regulation/law

Following the research implications regarding the strategic alignment of OGD programs and the corresponding OGD policy intensity, two research areas become apparent within which further research efforts can contribute to a better understanding of the specific context: the normative composition and implementation of OGD and the potential impacts of norms and policies. For the research area of normative composition and implementation of OGD it is stated that the far-reaching innovations for the state and the economy emerging from the implementation and use of open data in general and OGD in particular, require dedicated and appropriate policies from state authorities. Thus, Khurshid et al. (2019) state that in the future it will be important to understand the reasons for slow diffusion and a consequently weak adoption of general data policies at the organizational and individual levels. Furthermore, procedural metadata standards and general data quality standards should be preceded by further research (Máchová & Lněnička, 2017; Shepherd et al., 2019).

In addition to general overview studies, further in-depth analyses of applied standards and directives should be conducted in the future, which in turn will help to provide stronger guidelines for the development of data policies. Regarding the potential impacts of norms and OGD policies, further research is needed to determine how the formulation and implementation of data policies and normative guidelines affect other core aspects, such as subsequent use or the general contribution to success (Kurtz et al., 2019). Moreover, it is necessary to investigate, how specific policies that focus on the commercial value of OGD contain the risk of conflict with other open data values (Zuiderwijk et al., 2016). In order to identify and classify corresponding dependencies and consequences in this context, comparative and qualitative exploratory approaches are promising to derive conclusions from related policies and directives.

**Fig. 5:**
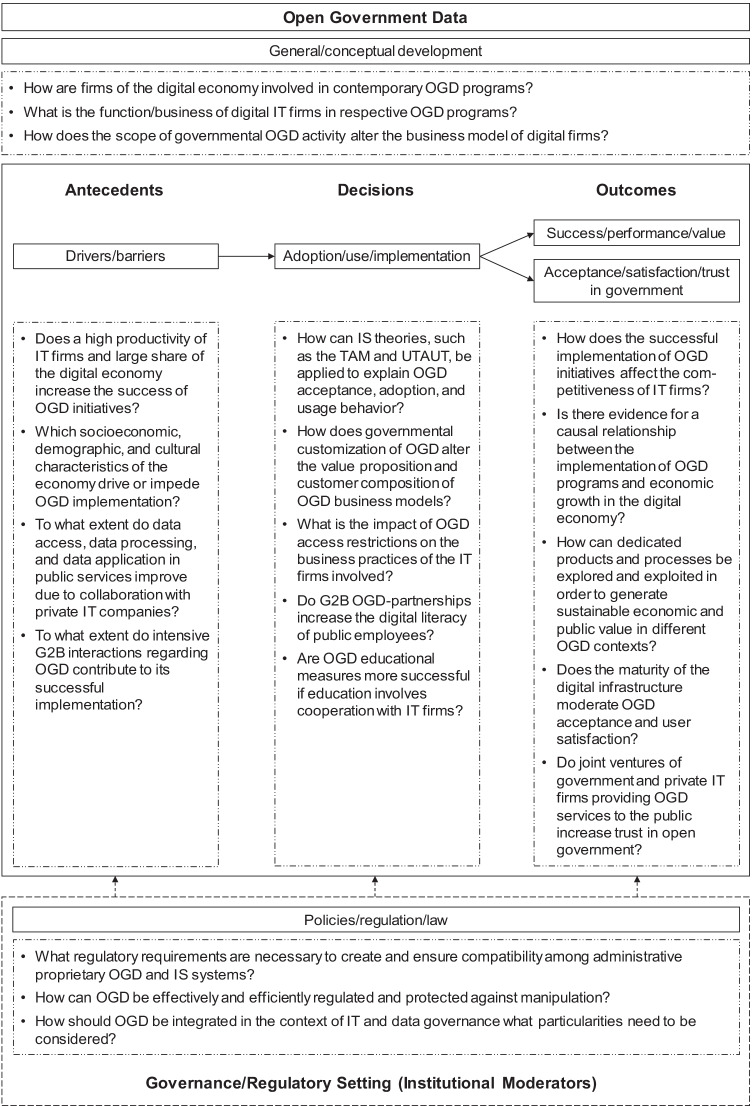
Theory-informed research agenda for IS and digital business research

In summary, a number of starting points for IS and digital business research emerge from the findings and insights of previous studies on the various OGD research areas. In the context of the consideration within the ADO framework, various parallels between the identified research questions also become apparent. To provide a general overview of these research implications, Fig. 5 reflects the relevant research questions and depicts their integration into the theoretical review framework in terms of a research agenda for IS and digital business research.

## Discussion and conclusion

Data have become an inherent part and essential driver of the digital economy. The field of OGD has been largely neglected by IS and digital business research, despite its great value potential for firms and the digital economy as a whole. As governments, public organizations, and firms worldwide are struggling in exploiting the full potential of OGD for the digital economy, it is essential to gain a comprehensive understanding of OGD and to frame the concept more broadly in the context of the digital economy in order to advance the field of research accordingly. On the one hand, this particularly requires greater involvement of the IS community in the very interdisciplinary field of OGD research, which is currently dominated by the public administration and public management perspective. On the other hand, it is necessary to theoretically integrate and synthesize the vast body of knowledge to identify research gaps and provide valid research directions.

An important requirement to achieve this is first and foremost conceptual clarity of OGD, which sometimes has been confounded with the related concepts of open government and open data. Our study goes beyond prior research (e.g., Hossain et al., 2016; Tai, 2021; Wirtz & Birkmeyer, 2015) by demonstrating and taking account of the – widely implicitly and tacitly assumed – conceptual autonomy of OGD and acknowledging it as an independent research stream closely related but still distinct from open government and open data research. This is a vital prerequisite for drawing differentiated and valid conclusions for the field and for gaining a clear understanding of the phenomenon. In this connection, we further build on and extend the general conceptual development of OGD and respective studies (e.g., Dawes et al., 2016; Kassen, 2013) by consolidating different OGD perspectives from the literature and by outlining the role of the digital economy in the OGD ecosystem and the digital economy’s relation to OGD-related government activity.

While previous research has made valuable contributions in structuring the OGD research landscape (e.g., Saxena, 2018; Zuiderwijk et al., 2014) and analysing certain OGD issues (e.g., Attard et al., 2015; Purwanto et al., 2020; Safarov et al., 2017), it fails to theoretically integrate the OGD concept and its key issues, and neglects the increasingly relevant relationship between OGD and the digital economy.

This study seeks to fill in this gap by conducting a systematic literature review of empirical OGD studies, which synthesizes the body of knowledge into a theoretical framework of OGD antecedents, decisions, and outcomes with special reference to the digital economy, and which further proposes a theory-informed research agenda for IS and digital business research.

Against this background, this study generally stands in line with and extends the findings of earlier comprehensive review approaches towards OGD literature, in particular those of Zuiderwijk et al. (2014) and Saxena (2018). However, these studies lack in the coherent linkage and the display of causal relationships between the different research areas as these studies mostly follow a descriptive approach attempting to present a common denominator of the characteristics of the individual studies. This study goes beyond their purely descriptive perspective by developing an overarching theoretical review framework that models the theoretical relationships of the thematic clusters identified in the literature analysis. In addition, this study also captures the more recent developments and novel empirical insights in the field of OGD. This is especially true for the area of OGD outcomes, for which research is based on a mature implementation of OGD systems in administrative practice, but also when it comes to issues such as organizational readiness and OGD skill development in the area of OGD decisions. Moreover, by examining the OGD literature with special reference to the digital economy, our study conceptually intersects with relevant IS and digital business research, demonstrating an interdisciplinary research approach that has been missing in prior OGD literature reviews.

Taken together, the theoretical attempt in conjunction with the focus on the digital economy and the associated inclusion of an IS perspective constitutes a new approach towards OGD literature that yielded novel insights into the field by integrating and explaining scientific progress in emergent topics such as in the areas of OGD decisions and OGD outcomes. Thus, the theoretical contribution of our study to the literature in terms of originality results from the theoretical review framework that theoretically integrates previously separated thematic clusters of OGD and their points of connection to IS and digital business research, thus improving our theoretical knowledge of the field of OGD and its relation to the digital economy. Overall, the synthesis of OGD literature into this theoretical framework represents the main response to our first research question of what we know about the antecedents, decisions, and outcomes of OGD and their relations in the context of the digital economy.

In this context, bridging the gap to digital business is of particular importance as this study represents the first attempt to transfer findings and insights from the mainly public administration- and public management-driven OGD studies to the IS and digital business research domains which might spark further progression in OGD research. The research agenda derived in accordance with the theoretical framework reveals how OGD research may relate to adjacent fields of IS and digital business research, such as interface design, IT and data governance, data security, big data analytics, open data, etc., and provides concrete opportunities and research questions in each thematic cluster.

Although the review provides valuable insights into each of the six key topics, the OGD outcomes appear to be of particular importance. This is not only indicated by the fact that this cluster already comprises the largest number of studies in relation to the other clusters, but also in view of very fundamental unresolved issues pertaining to the digital economy. We know today that the use of OGD opens up far-reaching opportunities for developing innovations and improving operational and business processes, for both the public and the private sector. Notwithstanding the awareness of those opportunities and increasing research on the potential benefits, the level of knowledge regarding how best to exploit and leverage economic value remains in many respects at incomplete (Magalhaes & Roseira, 2020; Ruijer & Meijer, 2020; Zuiderwijk et al., 2014). In particular in this context, but also in any of the other key topics, the research avenues identified indicate that OGD research may greatly benefit from the so far underrepresented IS and digital business perspective. As such it may serve as an important tool to build the bridge from OGD to IS and digital business research.

Overall, the research agenda synthesizes the answers to our second research question of how IS and digital business research can inform OGD research, in particular with regard to its role in the digital economy. The theoretical contribution of our study in terms of utility stems especially from the systematization of the complex and heterogeneous research landscape of OGD, as well as the theory-informed research agenda. The latter makes the field more accessible and tangible for IS and digital business research by showing what issues may be studied and how they are related.

However, our study is not without limitations. Merging information obtained from research databases bears a certain risk associated with information technology limitations and time delays that may prevent the full scope of relevant studies from being represented. In addition, our final sample is limited to studies in English language, which means that we may have missed potentially relevant studies in other languages. Bearing in mind that a complete selection is hardly feasible in terms of practicality and that the literature work on which this study is based was generated with respect to well-established methodological guidelines (Rowe, 2014; Webster & Watson, 2002), we are nevertheless convinced of the sufficient coverage and informative value provided by our relevant set. In addition, our analysis is limited to empirical studies and does not take account of conceptual approaches. Future research could examine whether the review framework also hold true in this connection and how empirical and conceptual OGD research differ in their distribution across the different key topics*.*

While our systematization and analyses enhance the level of lucidity and understanding with regard to the overall context of OGD, it should be noted that the six identified key topics require further dedicated attention in order to thoroughly interpret and understand the insights of the respective subareas. In this connection, it should further be noted that some of these topics have also been discussed in related research areas, in particular the more general field of open data, which have not been part of our literature review. Future research could synthesize these research streams and examine how they complement our findings. Finally, our comprehensive approach inherently goes at the expense of a detailed examination and discussion of each key topic. Although the majority of literature reviews on OGD have focused on a special key topic, it remains an important task for future studies to scrutinize recent, widely unexplored subtopics in OGD research, such as innovation and value creation.

In conclusion, although OGD has accumulated a substantial body of knowledge over the last decade, the field is still in an emerging stage and calls for further research to provide answers to a variety of important unresolved issues from an IS perspective. This systematic literature review contributes to a comprehensive understanding of OGD and may serve as a suitable reference point and impetus in bridging the gap between OGD and IS research and exploiting the potential of OGD for the digital economy.

## Supplementary information

ESM 1 (DOCX 39 kb)

## References

[CR1] Afful-Dadzie E, Afful-Dadzie A (2017). Open government data in Africa: A preference elicitation analysis of media practitioners. Government Information Quarterly.

[CR2] Alexopoulos C, Loukis E, Mouzakitis S, Petychakis M, Charalabidis Y (2018). Analysing the characteristics of open government data sources in Greece. Journal of the Knowldege Economy.

[CR3] Altayar, M. S. (2018). Motivations for open data adoption: An institutional theory perspective. *Government Information Quarterly, 35*(4), 633–643. 10.1016/j.giq.2018.09.006

[CR4] Attard J, Orlandi F, Scerri S, Auer S (2015). A systematic review of open government data initiatives. Government Information Quarterly.

[CR5] Bachtiar, A., Suhardi, & Muhamad, M. (2020). Literature review of open government data. *2020 International Conference on Information Technology Systems and Innovation (ICITSI)* (pp. 329–334).

[CR6] Barry E, Bannister F (2014). Barriers to open data release: A view from the top. Information Polity: The International Journal of Government & Democracy in the Information Age.

[CR7] Berrone, P., Ricart, J., & Carrasco-Farré, C. (2017). The open kimono: Toward a general framework for open data initiatives in cities. *California Management Review 59*(1), 39–70. 10.1177/0008125616683703

[CR8] Bogdanović-Dinić S, Veljković N, Stoimenov L, Rodríguez-Bolívar MP (2014). How open are public government data? An assessment of seven open data portals. Public administration and information technology. Measuring E-government efficiency.

[CR9] Bonina, C., & Eaton, B. (2020). Cultivating open government data platform ecosystems through governance: Lessons from Buenos Aires, Mexico City and Montevideo. *Government Information Quarterly, 37*(3). 10.1016/j.giq.2020.101479

[CR10] Borgesius FZ, Gray J, van Eechoud M (2015). Open data, privacy, and fair information principles: Towards a balancing framework. Berkeley Technology Law Journal.

[CR11] Callaway E, Ledford H, Viglione G, Watson T, Witze A (2020). COVID and 2020: An extraordinary year for science. Nature.

[CR12] Chatfield, A., & Reddick, C. (2017). A longitudinal cross-sector analysis of open data portal service capability: The case of Australian local governments. *Government Information Quarterly, 34*(2), 231–243.

[CR13] Chorley, K. M. (2017). The challenges presented to records management by open government data in the public sector in england. *Records Management Journal, 27*(2), 149–158. 10.1108/RMJ-09-2016-0034

[CR14] Criado JI, Ruvalcaba-Gómez EA, Valenzuela-Mendoza R (2018). Revisiting the open government phenomenon. A Meta-analysis of the international literature. JeDEM - eJournal of eDemocracy and Open Government.

[CR15] Crusoe J, Melin U, Parycek P, Glassey O, Janssen M, Scholl HJ, Tambouris E, Kalampokis E, Virkar S (2018). Investigating Open Government Data Barriers. Lecture notes in computer science. Electronic government.

[CR16] Dawes SS, Vidiasova L, Parkhimovich O (2016). Planning and designing open government data programs: An ecosystem approach. Government Information Quarterly.

[CR17] De Kool, D. & Bekkers, V. J. J. M. (2014). The perceived impact of open inspection data on the quality of education in Dutch primary schools: A parent perspective. *Social Science Computer Review*. 10.1177/0894439314560853

[CR18] Fan B, Zhao Y (2017). The moderating effect of external pressure on the relationship between internal organizational factors and the quality of open government data. Government Information Quarterly.

[CR19] Francey, A., & Mettler, T. (2021). The effects of open government data: Some stylised facts. Information Polity: *The International Journal of Government & Democracy in the Information Age,* 1–16. 10.3233/ip-200281.

[CR20] Gascó-Hernández, M., Martin, E. G., Reggi, L., Pyo, S., & Luna-Reyes, L. F. (2018). Promoting the use of open government data: Cases of training and engagement. *Government Information Quarterly, 35*(2), 233–242. 10.1016/j.giq.2018.01.003

[CR21] González-Zapata, F. & Heeks, R. (2015). The multiple meanings of open government data: Understanding different stakeholders and their perspectives. *Government Information Quarterly, 32*(4), 441–452. 10.1016/j.giq.2015.09.001

[CR22] Gonzálvez-Gallego, N., Nieto-Torrejón, L., & Pérez-Cárceles, M. (2020). Is open data an enabler for trust? Exploring the link and the mediating role of citizen satisfaction. *International Journal of Public Administration, 43*(14), 1218–1227. 10.1080/01900692.2019.1668412

[CR23] Gusenbauer M (2019). Google scholar to overshadow them all? Comparing the sizes of 12 academic search engines and bibliographic databases. Scientometrics.

[CR24] Haini SI, AB R, Mohd NZ, Zainuddin NM, Ibrahim R (2020). Factors influencing the adoption of open government data in the public sector: A systematic literature review. International Journal on Advanced Science, Engineering and Information Technology.

[CR25] Hassan, M. I. A., & Twinomurinzi, H. (2018). A systematic literature review of open government data research: Challenges, opportunities and gaps. *2018 Open Innovations Conference (OI)* (pp. 299–304).

[CR26] Hermanto, A., Solimun, S., Rinaldo Fernandes, A. A., Wahyono, W., & Zulkarnain, Z. (2018). The importance of open government data for the private sector and ngos in indonesia. *Digital Policy, Regulation and Governance, 20*(4), 293–309. https://search.ebscohost.com/login.aspx?direct=true&db=eoh&AN=1724283&site=ehost-live

[CR27] Hossain MA, Dwivedi YK, Rana NP (2016). State-of-the-art in open data research: Insights from existing literature and a research agenda. Journal of Organizational Computing & Electronic Commerce.

[CR28] Janssen M, Charalabidis Y, Zuiderwijk A (2012). Benefits, adoption barriers and myths of open data and open government. Information Systems Management.

[CR29] Jetzek, T., Avital, M., & Bjørn-Andersen, N. (2013). Generating value from open government data. In R. Baskerville, & M. Chau (Eds.), *Proceedings of the 34th International Conference on Information Systems (ICIS 2013)*. http://aisel.aisnet.org/cgi/viewcontent.cgi?article=1181&context=icis2013

[CR30] Jetzek, T., Avital, M., & Bjørn-Andersen, N. (2014). Data-driven innovation through open government data. *Journal of Theoretical and Applied Electronic Commerce Research, 9*(2), 100–120. 10.4067/S0718-18762014000200008

[CR31] Jetzek T, Avital M, Bjørn-Andersen N (2019). The sustainable value of open government data. Journal of the Association for Information Systems.

[CR32] Karkin N, Yavuz N (2017). An inquiry for local open government data policy: Is a proactive model of open government data portals possible in Turkey?. Current Politics and Economics of the Middle East.

[CR33] Kassen M (2013). A promising phenomenon of open data: A case study of the Chicago open data project. Government Information Quarterly.

[CR34] Kessler EH, Chakrabarti AK (1996). Innovation speed: A conceptual model of context, antecedents, and outcomes. Academy of Management Review.

[CR35] Khurshid MM, Zakaria NH, Rashid A, Kazmi R, Shafique MN, Nazir Ahmad M (2019). Analyzing diffusion patterns of big open data as policy innovation in public sector. Computers & Electrical Engineering.

[CR36] Kim H (2018). Interlinking open government data in Korea using Administrative District knowledge graph. Journal of Information Science Theory & Practice (JIStaP).

[CR37] Kleiman F, Meijer S, Janssen M (2020). Behavioral factors influencing the opening of government data by civil servants. In : *ICEGOV 2020, Proceedings of the 13th International Conference on Theory and Practice of Electronic Governance* (pp. 529–534).

[CR38] Kurtz LP, Santos PM, Rover AJ, Zuiderwijk A, Hinnant CC (2019). Open data via websites of Brazilian superior courts of justice: Changes between 2013 and 2017. Information Polity: The International Journal of Government & Democracy in the Information Age.

[CR39] Lane PJ, Koka BR, Pathak S (2006). The reification of absorptive capacity: A critical review and rejuvenation of the construct. Academy of Management Review.

[CR40] Lee SY, Díaz-Puente JM, Martin S (2019). The contribution of open government to prosperity of society. International Journal of Public Administration.

[CR41] Liberati A, Altman DG, Tetzlaff J, Mulrow C, Gøtzsche PC, Ioannidis JPA (2009). The PRISMA statement for reporting systematic reviews and meta-analyses of studies that evaluate health care interventions: Explanation and elaboration. PLoS Medicine.

[CR42] Lim, T. C. (2021). Patterns in environmental priorities revealed through government open data portals. *Telematics and Informatics, 101678*. 10.1016/j.tele.2021.101678

[CR43] Lim WM, Yap S-F, Makkar M (2021). Home sharing in marketing and tourism at a tipping point: What do we know, how do we know, and where should we be heading?. Journal of Business Research.

[CR44] Luna-Reyes LF, Najafabadi MM, Zuiderwijk A, Hinnant CC (2019). The US open data initiative: The road ahead. Information Polity: The International Journal of Government & Democracy in the Information Age.

[CR45] Máchová R, Lněnička M (2017). Evaluating the quality of open data portals on the National Level. Journal of Theoretical & Applied Electronic Commerce Research.

[CR46] Magalhaes, G., & Roseira, C. (2020). Open government data and the private sector: An empirical view on business models and value creation. *Government Information Quarterly, 37*(3). 10.1016/j.giq.2017.08.004

[CR47] Marjanovic, O. & Cecez-Kecmanovic, D. (2017). Exploring the tension between transparency and datification effects of open government IS through the lens of Complex Adaptive Systems. *The Journal of Strategic Information Systems, 26*(3), 210–232. 10.1016/j.jsis.2017.07.001

[CR48] Marmier A, Mettler T (2020). Developing an index for measuring OGD publisher compliance to good practice standards: Insights from opendata.Swiss. Information Polity: The International Journal of Government & Democracy in the Information Age.

[CR49] Martín-Martín A, Orduna-Malea E, Harzing A-W, Delgado López-Cózar E (2017). Can we use Google scholar to identify highly-cited documents?. Journal of Informetrics.

[CR50] Martín-Martín, A., Thelwall, M., Orduna-Malea, E., & Delgado López-Cózar, E. (2020). Google Scholar, Microsoft Academic, Scopus, Dimensions, Web of Science, and OpenCitations' COCI: A multidisciplinary comparison of coverage via citations. *Scientometrics,* 1–36. 10.1007/s11192-020-03690-4. 10.1007/s11192-020-03690-4 PMC7505221 32981987

[CR51] McBride K, Aavik G, Toots M, Kalvet T, Krimmer R (2019). How does open government data driven co-creation occur? Six factors and a 'perfect storm'; insights from Chicago's food inspection forecasting model. Government Information Quarterly.

[CR52] McNutt, J., Justice, J., Melitski, J., Ahn, M., Siddiqui, S., Carter, D., & Kline, A. (2016). The diffusion of civic technology and open government in the United States. *Information Polity, 21*(2), 153–170. 10.3233/IP-160385

[CR53] Meijer, A. (2015). Government transparency in historical perspective: From the ancient regime to open data in The Netherlands. *International Journal of Public Administration, 38*(3), 189–199. 10.1080/01900692.2014.934837

[CR54] Meijer A, Potjer S (2018). Citizen-generated open data: An explorative analysis of 25 cases. Government Information Quarterly.

[CR55] Mergel I, Kleibrink A, Sörvik J (2018). Open data outcomes: U.S. cities between product and process innovation. Government Information Quarterly.

[CR56] Neto AJA, Neves DF, Santos LC, Junior MCR, do Nascimento, Rogério P. C. (2018). Open government data usage overview: A systematic literature mapping. In : *EATIS ‘18, Proceedings of the Euro American Conference on Telematics and Information Systems*.

[CR57] Novais, T., Albuquerque, J. P. de, & Craveiro, G. S. (2013). An account of research on open government data (2007–2012): A systematic literature review. In Scholl H., Wimmer M.A., Tambouris E., Janssen M., & Macintosh A. (Eds.) (pp. 76–83). *Gesellschaft fur Informatik (GI)*. Retrieved from https://www.scopus.com/inward/record.uri?eid=2-s2.0-84918551984&partnerID=40&md5=8b722dd21af1f1c3743202eaeaae389d

[CR58] Open Knowledge Foundation. (2021). *Open definition: Defining open in open data, open content and open knowledge*. Retrieved from http://opendefinition.org/

[CR59] Parycek, P., Höchtl, J., & Ginner, M. (2014). Open Government Data Implementation Evaluation. *Journal of theoretical and applied electronic commerce research, 9*(2), 80–99. 10.4067/S0718-18762014000200007

[CR60] Paul J, Benito GRG (2018). A review of research on outward foreign direct investment from emerging countries, including China: What do we know, how do we know and where should we be heading?. Asia Pacific Business Review.

[CR61] Paul J, Criado AR (2020). The art of writing literature review: What do we know and what do we need to know?. International Business Review.

[CR62] Piotrowski SJ (2017). The “open government reform” movement. American Review of Public Administration.

[CR63] Purwanto A, Zuiderwijk A, Janssen M (2020). Citizen Engagement With Open Government Data. International Journal of Electronic Government Research.

[CR64] Raisch S, Birkinshaw J (2008). Organizational ambidexterity: Antecedents, outcomes, and moderators. Journal of Management.

[CR65] Roa, H. N., Loza-Aguirre, E., & Flores, P. (2019). A survey on the problems affecting the development of open government data initiatives. In Teran L., Meier A., & Pincay J. (Eds.) (pp. 157–163). *Institute of Electrical and Electronics Engineers Inc. *Retrieved from https://www.scopus.com/inward/record.uri?eid=2-s2.0-85068367066&doi=10.1109%2fICEDEG.2019.8734452&partnerID=40&md5=edfd3858943a1342f976f8bc20cf71e9

[CR66] Rowe F (2014). What literature review is not: Diversity, boundaries and recommendations. European Journal of Information Systems.

[CR67] Ruijer E, Grimmelikhuijsen S, Meijer A (2017). Open data for democracy: Developing a theoretical framework for open data use. Government Information Quarterly.

[CR68] Ruijer E, Martinius E (2017). Researching the democratic impact of open government data: A systematic literature review. Information Polity: The International Journal of Government & Democracy in the Information Age.

[CR69] Ruijer E, Meijer A (2020). Open government data as an innovation process: Lessons from a living lab experiment. Public Performance & Management Review.

[CR70] Safarov, I. (2019). Institutional dimensions of open government data implementation: Evidence from the Netherlands, Sweden, and the UK. *Public Performance & Management Review, 42*(2), 305–328. 10.1080/15309576.2018.1438296

[CR71] Safarov I, Meijer A, Grimmelikhuijsen S (2017). Utilization of open government data: A systematic literature review of types, conditions, effects and users. Information Polity: The International Journal of Government & Democracy in the Information Age.

[CR72] Saldaña J (2013). *The coding manual for qualitative researchers* (2. ed.).

[CR73] Saxena S (2018). Summarizing the decadal literature in open government data (OGD) research: A systematic review. Foresight.

[CR74] Saxena S, Janssen M (2017). Examining open government data (OGD) usage in India through UTAUT framework. Foresight.

[CR75] Sayogo, D. S., Pardo, T. A., & Cook, M. (2014). A framework for benchmarking open government data efforts. *47th Hawaii International Conference on System Sciences* (pp. 1896–1905). 10.1109/HICSS.2014.240

[CR76] Shao DD, Saxena S (2019). Barriers to open government data (OGD) initiative in Tanzania: Stakeholders' perspectives. Growth and Change.

[CR77] Shepherd E, Bunn J, Flinn A, Lomas E, Sexton A, Brimble S (2019). Open government data: Critical information management perspectives. Records Management Journal.

[CR78] Smith, G., & Sandberg, J. (2018). Barriers to innovating with open government data: Exploring experiences across service phases and user types. *Information Polity: The International Journal of Government & Democracy in the Information Age, 23*(3), 249–265. 10.3233/IP-170045

[CR79] Susha, I., Grönlund, Å., & Janssen, M. (2015). Driving factors of service innovation using open government data: An exploratory study of entrepreneurs in two countries. *Information Polity: The International Journal of Government & Democracy in the Information Age, 20*(1), 19–34. 10.3233/IP-150353

[CR80] Tai K-T (2021). Open government research over a decade: A systematic review. Government Information Quarterly.

[CR81] Tranfield D, Denyer D, Smart P (2003). Towards a methodology for developing evidence-informed management knowledge by means of systematic review. British Journal of Management.

[CR82] Ubaldi B (2013). Open government data: Open government data: towards empirical analysis of open government data initiatives. OECD working papers on public governance (No. 22).

[CR83] Urquhart C (2013). Grounded theory for qualitative research: A practical guide.

[CR84] Veljković N, Bogdanović-Dinić S, Stoimenov L (2014). Benchmarking open government: An open data perspective. Government Information Quarterly.

[CR85] Wang, H.‑J., & Lo, J. (2016). Adoption of open government data among government agencies. *Government Information Quarterly, 33*(1), 80–88. 10.1016/j.giq.2015.11.004

[CR86] Wang D, Chen C, Richards D (2018). A prioritization-based analysis of local open government data portals: A case study of Chinese province-level governments. Government Information Quarterly.

[CR87] Wang Y, Han JH, Beynon-Davies P (2019). Understanding blockchain technology for future supply chains: A systematic literature review and research agenda. Supply Chain Management: An International Journal.

[CR88] Wang, V., & Shepherd, D. (2020). Exploring the extent of openness of open government data – a critique of open government datasets in the UK. *Government Information Quarterly, 37*(1). 10.1016/j.giq.2019.101405

[CR89] Watson RT, Webster J (2020). Analysing the past to prepare for the future: Writing a literature review a roadmap for release 2.0. Journal of Decision Systems.

[CR90] Webster J, Watson RT (2002). Analyzing the past to prepare for the future: Writing a literature review. Management Information Systems Quarterly.

[CR91] Welch, E., Feeney, M., & Park, C. H. (2016). Determinants of data sharing in U.S. city governments. *Government Information Quarterly, 33*(3), 393–403. 10.1016/j.giq.2016.07.002

[CR92] Wirtz BW, Birkmeyer S (2015). Open government: Origin, development, and conceptual perspectives. International Journal of Public Administration.

[CR93] Wirtz, B. W., Weyerer, J. C., & Rösch, M. (2018). Citizen and open government: An empirical analysis of antecedents of open government data. *International Journal of Public Administration, 41*(4), 308–320. 10.1080/01900692.2016.1263659

[CR94] Wirtz BW, Weyerer JC, Rösch M (2019). Open government and citizen participation: An empirical analysis of citizen expectancy towards open government data. International Review of Administrative Sciences.

[CR95] World Wide Web Foundation. (2017). *Open data barometer: Global report*. Retrieved from https://opendatabarometer.org/doc/4thEdition/ODB-4thEdition-GlobalReport.pdf

[CR96] Worthy, B. (2015). The impact of open data in the UK: Complex, unpredictable, and political. *Public Administration, 93*(3), 788–805. 10.1111/padm.12166

[CR97] Yang, T.-M., Lo, J., & Shiang, J. (2015). To open or not to open? Determinants of open government data. *Journal of Information Science, 41*(5), 596–612. 10.1177/0165551515586715

[CR98] Yang, T. M., & Wu, Y. J. (2016). Examining the socio-technical determinants influencing government agencies’ open data publication: A study in Taiwan. *Government Information Quarterly, 33*(3):378–392. 10.1016/j.giq.2016.05.003

[CR99] Young MM (2020). Implementation of digital-era governance: The case of open data in U.S Cities. Public Administration Review.

[CR100] Zhao, Y., & Fan, B. (2018). Exploring open government data capacity of government agency: Based on the resource-based theory. *Government Information Quarterly, 35*(1), 1–12. 10.1016/j.giq.2018.01.002

[CR101] Zhenbin Y, Kankanhalli A, Ha S, Tayi GK (2020). What drives public agencies to participate in open government data initiatives? An innovation resource perspective. Information & Management.

[CR102] Zientek LR, Werner JM, Campuzano MV, Nimon K (2018). The use of Google scholar for research and research dissemination. New Horizons in Adult Education and Human Resource Development.

[CR103] Zuiderwijk A, Helbig N, Gil-Garcia J, Janssen M (2014). Innovation through open data: A review of the state-of-the-art and an emerging research agenda. Journal of Theoretical & Applied Electronic Commerce Research.

[CR104] Zuiderwijk, A., Janssen, M., & Dwivedi, Y. K. (2015). Acceptance and use predictors of open data technologies: Drawing upon the unified theory of acceptance and use of technology. *Government Information Quarterly, 32*(4), 429–440. 10.1016/j.giq.2015.09.005

[CR105] Zuiderwijk A, Janssen M, van de Kaa G, Poulis K (2016). The wicked problem of commercial value creation in open data ecosystems: Policy guidelines for governments. Information Polity: The International Journal of Government & Democracy in the Information Age.

