# Supplementary material for: Open government data: A systematic literature review of empirical research
Source: Electron Mark. 2022 Sep 20;32(4):2381–404. doi: 10.1007/s12525-022-00582-8 (PMC9487844; doi:10.1007/s12525-022-00582-8)
Supplement: Supplementary file 1 — (DOCX 39 kb) [file 12525_2022_582_MOESM1_ESM.docx]

**Online Appendix
Table 1: Findings of Previous Research with regard to the OGD ADO-Framework**

|  | **Research Area** | **Key Findings** | **Most relevant studies** |
| --- | --- | --- | --- |
| **OGD Antecedents** | **Drivers** | **OGD drivers / catalysts / enablers (factors triggering OGD as a government policy)**   - **Political and social OGD drivers:** refer to those drivers of OGD programs that emerge from political pressure upon or social attitudes towards the government and state institutions including (1) political demand for transparency and government accountability (Zhenbin et al. 2020; Barry and Bannister 2014; Janssen et al. 2012), (2) increased citizen involvement and participation in state affairs (Young et al. 2020; Welch et al. 2016; Janssen et al. 2012), (3) widespread use of ICT devices amongst the population relying upon data for providing online services and apps to its users (Young 2020; Smith and Sandberg 2018), and (4) informatization and innovativeness of society expressing a public need for public sector data due to societal curiosity (Smith and Sandberg 2018; Susha et al. 2015). - **Operational and technical OGD drivers:** refer to those drivers of OGD programs that emerge from digitization and digitalization of public administration including (1) commitment of public agency culture to electronic data processing and data sharing (Zhenbin et al. 2020; Yang et al. 2015), (2) degree of digitization of applications, forms, and reports (Young 2020; Yang et al. 2015), (3) degree of process and infrastructure digitalization within the agency (Susha et al. 2015), and (4) staff skills and training in data handling (Welch et al. 2016; Janssen et al. 2012) - **Economic OGD drivers:** refer to those drivers of OGD programs that emerge from the business opportunities being realized by the commercialization of government data including (1) moderate to high share of privately operated public utilities (e.g. hospitals, energy and water suppliers, waste collection, etc.) (Young 2020; Susha et al. 2015), (2) increased demand and high prices paid for public data licenses for offering public online services to the citizens (Zhenbin et al. 2020), and (3) high levels of GDP in general (high income) and moderate to high share of the ICT sector in the national economy (high productivity of ICT) (Young 2020; Susha et al. 2015). | Young 2020  Zhenbin et al. 2020  Smith & Sandberg 2018  Ruijer et al. 2017  Welch et al. 2016  Susha et al. 2015  Yang et al. 2015  Barry & Bannister 2014  Conradie & Choenni 2014  Janssen et al. 2012 |
|  | **Barriers** | **OGD barriers / impediments / constraints (factors impeding OGD as a government policy)**   - **Data compilation constraints (institutional barriers):** refer to OGD barriers emerging from institutional/organizational barriers to data compilation and data provision (Ruijer et al. 2017; Barry and Bannister 2014; Conradie and Choenni 2014). Previous research differentiates three major obstacle clusters associated with data compilation constraints: (1) legal barriers to OGD such as data protection/privacy violation, restrictions in data confidentiality, or opaqueness of data ownership (Yang et al. 2015; Barry and Bannister 2014; Conradie and Choenni 2014), (2) structural barriers to OGD such as high data fragmentation and decentralization, degree of information system outsourcing, or complex hierarchical structures (Ruijer et al. 2017; Welch et al. 2016; Yang et al. 2015), or (3) capability barriers to OGD such as a lack in data management capabilities and capacities, budget and resource constraints, or lack of statistical staff for data processing and preparation (Young 2020; Ruijer et al. 2017; Janssen et al. 2012). - **Data access constraints (technical barriers):** refer to OGD barriers emerging from the properties of the underlying system providing data to the citizens (Smith and Sandberg 2018; Ruijer et al. 2017; Barry and Bannister 2014). As major properties of the system impeding the implementation of OGD, previous studies identify (1) lack of system interoperability due to a fragmentation in processing software or non-standardized data formats (Smith and Sandberg 2018; Ruijer et al. 2017; Barry and Bannister 2014), (2) lack of technical support for data platforms and APIs due to shortages in ICT staff leading to slow data provision and low back-end reliability (Smith and Sandberg 2018; Janssen et al. 2012), and (3) access constraints by obligatory user accounts or official online registration for data access (Ruijer et al. 2017; Barry and Bannister 2014). - **Data application constraints (societal barriers):** refer to OGD barriers emerging from socio-economic characteristics of the resident population (such as demographics, literacy rate, education level, private ICT equipment expenditure, technological affinity, etc.) (Young 2020; Welch et al. 2016). Hence this, previous literature argues that barriers stemming from the inability of the citizens to apply publicly provided data states a severe barrier for OGD programs (Ruijer et al. 2017; Janssen et al. 2012), This inability might either appear due to a lack in citizen information processing, i.e. data cannot be processed by the citizens (Ruijer et al. 2017; Barry and Bannister 2014) or due to the lack of practical use of the data for the relevant target groups, i.e. the citizens do not know what to do with these data (Smith and Sandberg 2018; Janssen et al. 2012). |  |

|  | **Research Area** | **Key Findings** | | **Most relevant studies** |
| --- | --- | --- | --- | --- |
| **OGD Decisions** | **OGD Adoption** | Decisions regarding the adoption of OGD programs include the specification of the overall OGD approach, policy, and strategy (Gascó-Hernandez et al. 2018; Dawes et al. 2016; Wang and Lo 2016) as well as organizational and technical preparations to get public administration ready for opening government data to the public (Chatfield and Reddick 2017; Yang and Wu 2016; Parycek et al. 2014): | | Bonina & Eaton 2020  Safarov 2019  Talukder et al. 2019  Wirtz et al. 2019  Altayar 2018  Gasco-Hernandez et al. 2018  Smith & Sandberg 2018  Wirtz et al. 2018  Chatfield & Reddick 2017  Dawes et al. 2016  Wang & Lo 2016  Yang & Wu 2016  Parycek et al. 2014 |
|  |  | **OGD policy and strategy / OGD policy intensity:**   - Data-oriented OGD: (Wang & Lo 2016; Yang & Wu 2016)   OGD efforts concentrate on the provision of ‘proper’ unprocessed datasets to the citizens or other agencies especially emphasizing “completeness, primacy, timeliness, ease of physical and electronic access, machine readability, and licensing” (Dawes et al. 2016, p. 17).   - Program-oriented OGD: (Chatfield & Reddick 2017; Parycek et al. 2014)   OGD efforts concentrate on a smooth function of data distribution channels such as OGD platforms. The focus is on tracking data publication processes in terms of “data content, manipulation capability, and participatory capability […] to better link data publication decisions  with public needs for data” (Dawes et al. 2016, p. 18).   - Use- and User-oriented OGD: (Gascó-Hernandez et al. 2018)   OGD efforts concentrate on providing public/social/economic value to  the citizens by using public sector data. Public administration thus provides preprocessed data via services or apps realizing a certain benefit to the citizen by applying the information to a specific problem. | **Organizational readiness:**   - Scope of OGD permission: (Wang & Lo 2016; Yang & Wu 2016)   Decisions that define the scope for decision-making for the implementing authority granted from high-level authorities; the focus is set on the type and the extent of data available to users, as well as the options for data handling granted to the citizens; the scope ranges from mere publication permissions to the support of an interactive data service.   - Maturity of data infrastructure: (Bonina & Eaton 2020; Wang & Lo 2016)   Decisions that define the state of the technical infrastructure to collect, process, and publish data in their respective field of employment; the focus is set on (1) the scope of hardware used for data management (technical capacity), and (2) the interoperability with ICT systems of other agencies, IT companies, or citizen devices (connectivity).   - State of data management capability: (Safarov 2019; Yang & Wu 2016)   Decisions that define the state of the ability of public administration to collect, process, and publish data in their respective field of employment; the focus is set on (1) the scope of software used for data management (technical capability), and (2) the skills and expertise of agency employees available for data management and analysis (staff capability). |  |
|  | **OGD Implementation and OGD Usage** | Decisions regarding the implementation of OGD programs and the manner of OGD usage include the creation of relevant skills and technical expertise in data handling (Gascó-Hernandez et al. 2018; Wirtz et al. 2018; Yang and Wu 2016) as well as the features, the design, and the target user profile of the OGD interface (Chatfield & Reddick 2017; Parycek et al. 2014): | |  |
|  |  | **Relevant skills and educational support:**   - OGD skill development: (Safarov 2019; Yang & Wu 2016)   Short- to medium-term educational measures to increase public  employees’ digital literacy and ability of handling data and associated  data applications in public administration and citizen services; decisions  concerning the extent of training is crucial, ranging from internal IT training (Yang & Wu 2016) to cooperation with private IT firms (Safarov 2019).   - Public IT schooling: (Gascó-Hernandez et al. 2018; Wirtz et al. 2018)   Long-term educational measures to increase the population’s general  knowledge about IT and data handling thereby increasing the initial  experience in data management of new public employees; decisions  appear stepwise starting with cooperation programs for open data  management at administrative science universities, continuing with the  creation of study programs focusing OGD in public administration, ending  up with the establishment of public data management schools (Gascó-  Hernandez et al. 2018, pp. 237-240). | **Interface design, features, and user profile**   - Target group for OGD: (Smith & Sandberg 2018; Parycek et al. 2014)   Decision that defines the stakeholder group targeted by the OGD program to customize data access and data manipulation options; previous studies differentiate three approaches for OGD customization: (1) general access for average citizens retrieving data for informative education and knowledge, (2) licensed access for commercial users retrieving data for providing augmented or new goods and services to their customers, and (3) specialist access for professional users retrieving data for creating expertise and innovation (cf. Smith & Sandberg, p. 257)   - Interface design and OGD features: (Chatfield & Reddick 2017)   The shape of the OGD interface is the result of three decisions: (1) decision on the amount and format of data opened for public access (scope of dataset), (2) decision on the shape of data access channels, i.e. website, portal, or platform (scope of data interface), and (3) decision on the scope of data analysis tools and data-based applications available on the OGD interface (scope of data functions). |  |

**Online Appendix
Table 1: (continued)**

|  | **Research Area** | **Key Findings** | | **Most relevant studies** |
| --- | --- | --- | --- | --- |
| **OGD Outcomes and Impacts** | **OGD Outcomes** | **Internal effects achieved by OGD implementation:**  **public administration performance**   - Efficiency gains in administrative processes (economic effects):   refer to the effects of OGD upon the performance of administrative procedures and of the provision of public services; gains in efficiency and effectives result from (1) more effective and targeted administrative action due to higher information quality, (2) reduced waiting times and shortened service routes for database queries, and (3) simplification of inter-agency cooperation due to mutual data exchange and data integration (Mergel et al. 2018; Worthy 2015)   - Transparency of political decision-making (accountability effects):   refer to the effects of OGD upon political accountability and traceability of political decision-making due to (1) information reconciliation between politicians, public employees and citizens by OGD, and (2) increase in duration and complexity of political decision-making processes due to participation of citizens via OGD tools (Marjanovic & Cecez-Kecmanovic 2017; Jetzek et al. 2014); however, the overall effect of OGD upon transparency remains controversial as the government is able (1) to provide data on neutral or irrelevant topics, and (2) to provide false data without any data verification (Wang & Shepherd 2020)   - Behavioral effects due to continuous recording (datafication effects):   refer to the effects of OGD upon the behavior of the public employees and policy-makers; these effects include (1) data-based discrimination, (2) misjudgement due to false causality assumption, (3) conformity pressure to prevent dataset "falsification", (4) excessive performance pressure to provide the "right" data or a good performance from the citizens' point of view, and (5) increased incentives for data manipulation (Marjanovic & Cecez-Kecmanovic 2017; Worthy 2015). | **External effects achieved by OGD implementation:**  **OGD success and public value**   - Citizen engagement and information exchange (interaction effects):   refer to the effects of OGD upon the participation and involvement of citizens into political affairs; previous studies find OGD to spark (1) more controversies in political debates due to improved information status of citizens and improved opportunities to track political decisions, (2) public service innovation due to feedback from citizens and experts outside of public administration, and (3) efficiency and effectiveness of NGOs and citizen initiatives by synergies created from data sharing (Ruijer & Meijer 2020; Máchová & Lnenicka 2017; Worthy 2015; Jetzek et al. 2014)   - Reduction of information asymmetries (information effects):   refers to the effects of OGD upon unequal distribution of information among the government (agent) and the citizens as sovereigns (principals); removing information asymmetries leads (1) to a power shift from the government to the citizens, i.e. democratization of public administration, and (2) to increased awareness of political compliance reducing corruption and public maladministration; these effects are counterbalanced as (1) the government decides upon the amount and type of data published, and as (2) the citizens need the ability to process OGD retrieving the required information (Wang & Shepherd 2020; Marjanovic & Cecez-Kecmanovic 2017; Jetzek et al. 2014)   - Creation of new public services (commercialization/innovation effects):   refers to the spillover effects of OGD upon the digital economy and the offering of data-based public online services; implementing OGD provides digital firms with the opportunity of (1) accessing a new resource, i.e. public sector data, for value creation, and (2) opening up new customer groups such as public employees or recipients of public services (Jetztek et al. 2019; Mergel et al. 2018; Jetztek et al. 2014) | Wang & Shepherd 2020  Jetzek et al. 2019  Ruijer & Meijer 2020  Mergel et al. 2018  Marjanovic & Cecez-Kecmanovic 2017  Máchová & Lnenicka 2017  Worthy 2015  Charalabdis et al. 2014  Jetzek et al. 2014 |
|  | **OGD Impacts** | **Consequences triggered by OGD implementation: Open data technology acceptance and citizen satisfaction / trust in government** | | Grimmelikhuisen et al. 2020  Gonzálvez-Gallego et al. 2020  Afful-Dadzie & Afful-Dadzie 2017  Ohemeng & Ofosu-Adarkwa 2015  Zuiderwijk et al. 2015  Cucciniello et al. 2015  DeKool & Bekkers 2015 |
|  |  | - Open data technology acceptance and citizen satisfaction:   refers to the impact of OGD upon the acceptance and usage of ICT in the context of public services as well as the impact of OGD upon the citizens’ satisfaction regarding government performance; a positive impact of OGD upon technology acceptance relies upon (1) sufficiently intense internet usage among the population (Gonzálvez-Gallego et al. 2020; Afful-Dadzie & Afful-Dadzie 2017), (2) awareness of individual benefits resulting from the use and application of OGD (Zuiderwijk et al. 2015; De Kool & Bekkers 2014), and (3) degree of OGD usage obligation when consulting public institutions or making use of public services (Gonzálvez-Gallego et al. 2020; Zuiderwijk et al. 2015); the positive impact of OGD upon citizen satisfaction depends upon (1) broad acceptance and support of OGD technologies among the population and (2) sufficiently high data and information quality as well as sufficiently high OGD service quality (Gonzálvez-Gallego et al. 2020). | - Trust in government:   refers to the impact of OGD upon the citizens’ trust in government and their authorities; a positive impact is realized if (1) public administration and the government provide OGD upon the topics relevant to the addressed stakeholders (Cucciniello et al. 2015), (2) the respective nation exhibits a comparatively high level of public order and political stability (Grimmelikhuisen et al. 2020), and (3) broad acceptance and support of OGD technologies among the population as well as high satisfaction with the information and system quality of OGD services (Gonzálvez-Gallego et al. 2020) |  |

**Online Appendix
Table 1: (continued)**
